# Local and long-distance organization of prefrontal cortex circuits in the marmoset brain

**DOI:** 10.1016/j.neuron.2023.04.028

**Published:** 2023-05-16

**Authors:** Akiya Watakabe, Henrik Skibbe, Ken Nakae, Hiroshi Abe, Noritaka Ichinohe, Muhammad Febrian Rachmadi, Jian Wang, Masafumi Takaji, Hiroaki Mizukami, Alexander Woodward, Rui Gong, Junichi Hata, David C. Van Essen, Hideyuki Okano, Shin Ishii, Tetsuo Yamamori

**Affiliations:** 1 Laboratory for Molecular Analysis of Higher Brain Function, RIKEN Center for Brain Science, Wako, Saitama 351-0198, Japan; 2 Laboratory for Haptic Perception and Cognitive Physiology, RIKEN Center for Brain Science, Wako, Saitama 351-0198, Japan; 3 Brain Image Analysis Unit, RIKEN Center for Brain Science, Wako, Saitama 351-0198, Japan; 4 Integrated Systems Biology Laboratory, Department of Systems Science, Graduate School of Informatics, Kyoto University, Kyoto, Kyoto 606-8501, Japan; 5 Exploratory Research Center on Life and Living Systems, National Institutes of Natural Sciences, Okazaki, Aichi 444-8787, Japan; 6 Department of Ultrastructural Research, National Institute of Neuroscience, National Center of Neurology and Psychiatry, Kodaira, Tokyo 187-0031, Japan; 7 Faculty of Computer Science, Universitas Indonesia, Depok, Jawa Barat 16424, Indonesia; 8 Division of Genetic Therapeutics, Center for Molecular Medicine, Jichi Medical University, Shimotsuke, Tochigi 329-0498, Japan; 9 Connectome Analysis Unit, RIKEN Center for Brain Science, Wako, Saitama 351-0198, Japan; 10 Laboratory for Marmoset Neural Architecture, RIKEN Center for Brain Science, Wako, Saitama 351-0198, Japan; 11 Graduate School of Human Health Sciences, Tokyo Metropolitan University, Tokyo 116-8551, Japan; 12 Department of Neuroscience, Washington University School of Medicine, Saint Louis, MO 63110, USA; 13 Department of Physiology, Keio University School of Medicine, Tokyo 108-8345, Japan; 14 Department of Marmoset Biology and Medicine, Central Institute for Experimental Animals, Kawasaki, Kanagawa 210-0821, Japan; 15 Lead contact

## Abstract

The prefrontal cortex (PFC) has dramatically expanded in primates, but its organization and interactions with other brain regions are only partially understood. We performed high-resolution connectomic mapping of the marmoset PFC and found two contrasting corticocortical and corticostriatal projection patterns: “patchy” projections that formed many columns of submillimeter scale in nearby and distant regions and “diffuse” projections that spread widely across the cortex and striatum. Parcellation-free analyses revealed representations of PFC gradients in these projections’ local and global distribution patterns. We also demonstrated column-scale precision of reciprocal corticocortical connectivity, suggesting that PFC contains a mosaic of discrete columns. Diffuse projections showed considerable diversity in the laminar patterns of axonal spread. Altogether, these fine-grained analyses reveal important principles of local and long-distance PFC circuits in marmosets and provide insights into the functional organization of the primate brain.

## In brief

In this article, Watakabe et al. perform extensive tracer mapping of the marmoset PFC, finding two types of projections (patchy and diffuse) to be topographically arranged in the cortex and striatum. Fine-grained analyses enabled by this new resource deepen our understanding of local and long-range connectivity of the primate PFC.

## Graphical Abstract

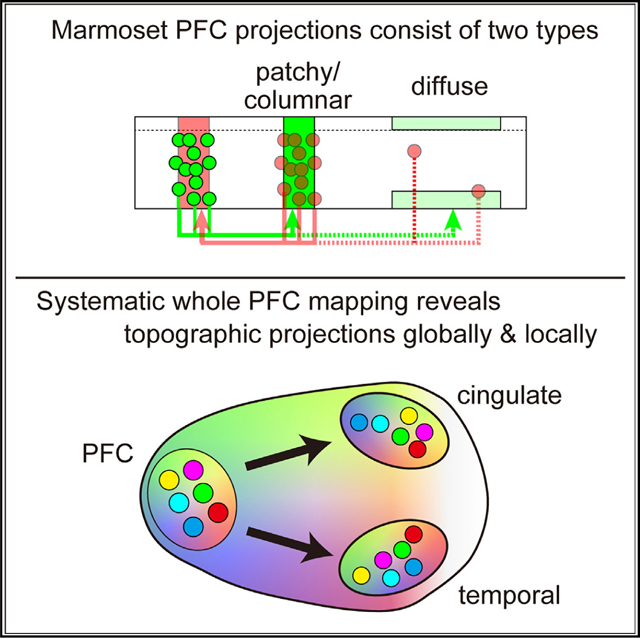

## INTRODUCTION

The prefrontal cortex (PFC) plays a central role in orchestrating the activities of various brain regions. The cognitive and emotional functions of the PFC enable complex behaviors, and its malfunction contributes to diverse mental disorders.^1,2^ PFC contains a mosaic of distinct cortical areas, with an estimated 45 PFC areas out of 180 total neocortical areas in humans^3^ and 35 PFC areas out of 130 total in macaques,^4^ and the marmoset has 26 PFC areas out of a 117-area parcellation.^5^ These areas have complex patterns of connections with many cortical and subcortical regions.^1,2,6,7^ However, our knowledge of PFC connectivity remains fragmentary, particularly for quantitative connectivity data of primates. An existing retrograde tracer database covers most but not all PFC subregions,^6,8^ and no quantitative analysis has been reported using anterograde tracers.

In primates, columnar organization has been studied extensively, particularly in the visual cortex. It generally reflects commonalities along the radial axis (from white matter to pia), exemplified by orientation columns and ocular dominance columns in area V1 and tuning for other features in extrastriate visual areas.^9–11^ Patchy anatomical connections of these columnar modules in the tangential domain correlate with repeating representations of various features such as stimulus orientation or eye dominance.^9,10^ However, because preferred orientation is represented as a smoothly changing variable, a single orientation “column” in visual cortex is more a conceptual abstraction than a discretely demarcated three-dimensional (3D) cortical domain. By contrast, submillimeter-scale discrete projections suggestive of a different type of columnar organization have been reported using anterograde tracer injections in macaque PFC, but major questions remain as to whether such patterns reflect discrete, segregated modules vs. highly overlapping connectivity profiles, whether they are predominantly patchy vs. stripe-like, and whether their origins and terminations are consistently columnar or are often layer-specific.^12–16^

In this study, we provide evidence bearing on these and other issues using a dataset based on 44 anterograde and 13 retrograde tracer injections into PFC and adjacent frontal lobe regions in common marmosets. The marmoset is an increasingly popular non-human primate (NHP) model for neuroscience studies.^17–24^ Its cortex is one-tenth the size and far less convoluted than the macaque cortex. Yet it contains the frontal eye field (FEF), V5 (middle temporal area [MT]), and granular PFC, all common to primates but lacking clearly defined homologs in rodents.^1,22,23^ Systematic analysis of our high-quality dataset revealed patchy and columnar corticocortical and corticostriatal axonal projections plus a complementary pattern of diffuse projections generally restricted to one or a few layers. By combining anterograde and retrograde double-tracing in some animals, we demonstrated a striking reciprocity of patchy cortical connectivity. We also mapped the topographic organization of PFC connectivity to multiple regions of the association cortex, revealing a pattern similar but not identical to that demonstrated by stimulation-fMRI mapping of macaque lateral PFC.^25^ Our datasets are freely accessible (https://dataportal.brainminds.jp/; see also Skibbe et al.^26^) and add to a growing collection of publicly available marmoset neuroscience-related datasets.^27^

## RESULTS

### STPT imaging in the marmoset brain

**Figure 1.**
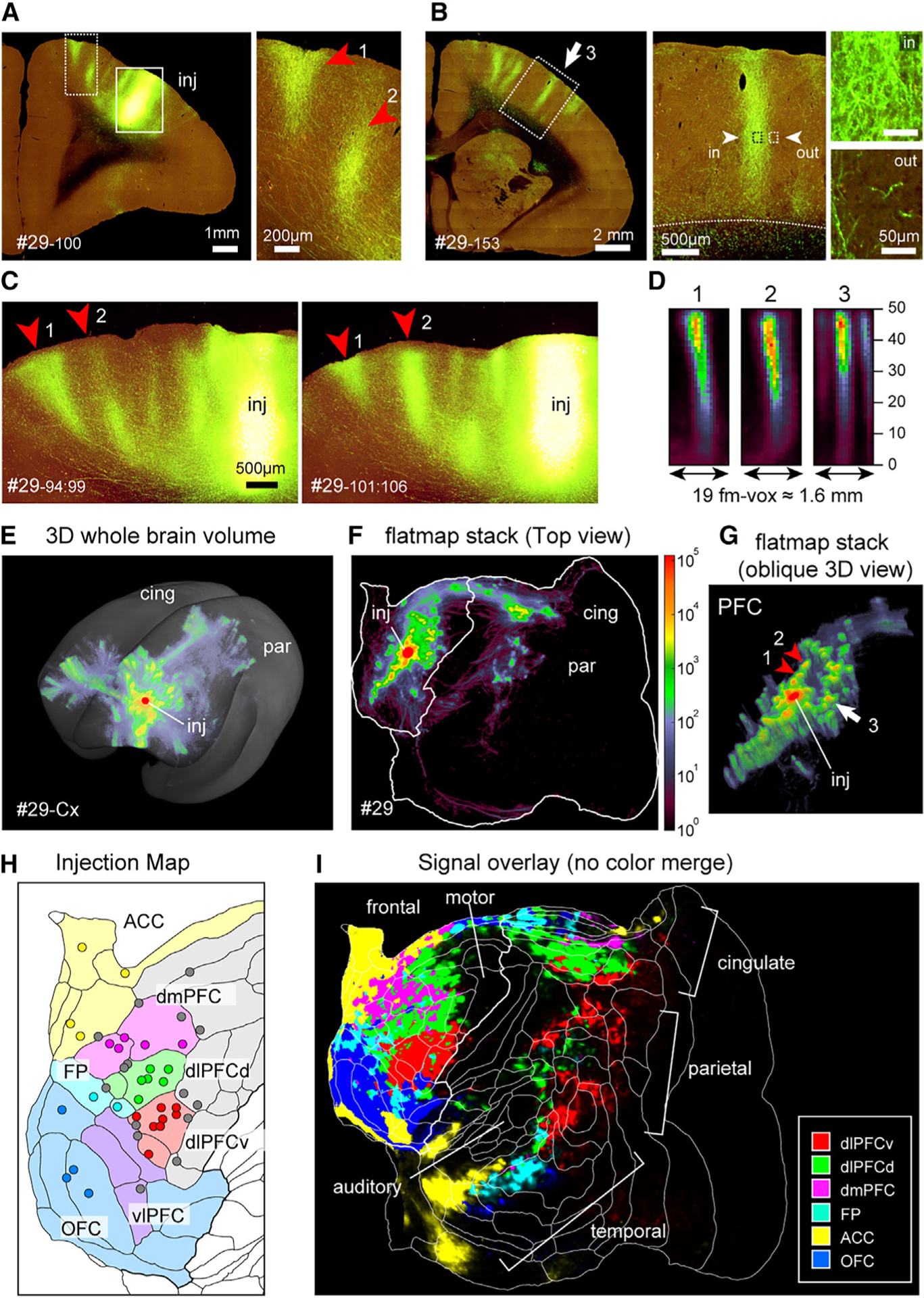
Patchy/columnar projections from marmoset PFC imaged in 3D (A) Original section image (#29–100 indicates section 100 of case #29) around the injection site (inj), cut slightly oblique to the radial axis. Note that we could identify the AAV-transduced cell bodies in the unsaturated channel (Figure S1D). Two red arrowheads (1 and 2) correspond to those in (C) and (G). (B) Another example of a columnar axonal convergence (white arrow 3) shown at three different magnifications of a section cut mostly parallel to the radial axis. (C) Maximum intensity projection (MIP) views for six serial slices showing radial extensions of columns 1 and 2, before and after section 100. (D) Three-dimensional (3D) reconstruction of columns 1, 2, and 3 in the flatmap stack shown as a MIP side view. This and other similar images (e.g., E–G) use the positive half of the “Videen” palette for pseudocolor intensity scaling (see STAR Methods). fm-vox, flatmap-voxel (see STAR Methods). (E) 3D reconstruction of the cortical tracer signals in the STPT template space. The log value and pseudocolor intensity scaling are used to show both strong columnar protrusions and diffuse spread over broad regions of the cortex. The injection site (inj) is shown by a red dot. cing, cingulate field; par, parietal field. (F) An MIP top view of the flatmap stack image for the left cortex for the data shown in (E). (G) A 3D view of the flatmap stack around the injection site. Example columnar projections (1–3) are indicated by the arrow and arrowheads. (H) Summary figure of the injections analyzed in this study (also see Figure S1G). Injections centered well inside a core domain (darker colors) were used for analyses of regional differences in connectivity. Injection sites shown in gray were excluded from this regional analysis either because they position at the boundary or in the premotor (PM) areas but were included in other parcellation-free analyses. (I) Overlay image of projections showing a systematic distribution pattern (linear scale and no color merge; see also Figures S1H and S1I). Subregion abbreviations: dorsolateral PFC-dorsal (dlPFCd), green; dorsolateral PFC-ventral (dlPFCv), red; dorsomedial PFC (dmPFC), magenta; frontopolar cortex (FP), cyan; anterior cingulate cortex (ACC), yellow; orbitofrontal cortex (OFC), blue. See also Figures S1–S3.

**Figure 2.**
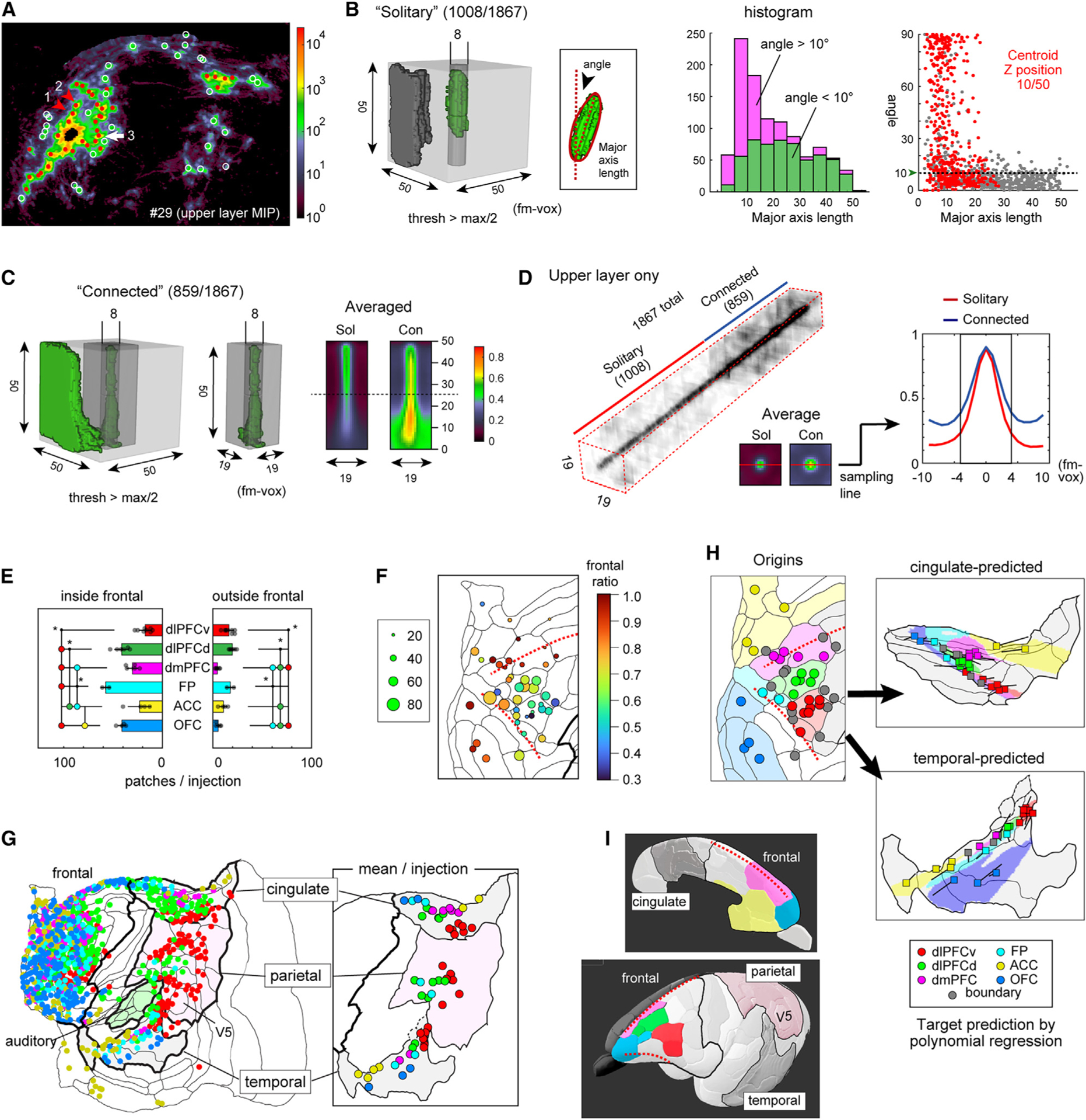
Topographic distribution of patchy cortical projections from PFC subregions (A) Detection of columnar patches in the cortical flatmap stack as local maxima. The red dots and white circles indicate patches with peak values that are >1/10 (10^3^) and >1/100 (10^2^) of the standardized peak value (10^4^), respectively. (B) 3D reconstruction of binarized tracer signals. We identified patches as connected or solitary based on whether the clump was (C) or was not (B) connected to surrounding labeled regions (see STAR Methods for details). The clumps for solitary patches were approximated by an ellipsoid, whose major axis length and angle of deviation were measured. The histogram shows the distribution of major axis lengths of ellipsoids with small (<10°; green) and large (>10°; magenta) angles. The red dots in the scatter plot show the clumps centered in the uppermost (10/50) compartment. fm-vox; flatmap-voxel (see STAR Methods). (C) An example of the connected patches. The averaged laminar profiles for the solitary (Sol) and the connected (Con) patches show the averages of the MIP view of the central 4 (thickness) × 19 (width) × 50 (depth) voxel boxes. (D) The non-binarized original tracer signals in the upper layer compartments were averaged to show their 19 × 19-fm-vox planar spreads for 1,867 columnar patches, which were aligned in 3D to show the central convergence. These planar images were further averaged for the two groups (Sol and Con) and the intensities were measured along the red sampling lines. (E) The average number of columnar patches per injection for six PFC subregions. The colored circles indicate statistically significant differences (ANOVA with Tukey’s honest significant difference test, p < 0.05) between the subregions of these colors and the subregions left (or right) of the circles. Data are represented as mean ± SD. (F) The number of columnar patches (in circle diameter) and the ratio of the frontal/total patch numbers (shown by color scale) for each injection are displayed. The red dotted lines indicate the dorsomedial and ventrolateral convexities (ridges) of the cortex (see I). (G) Distribution of the columnar patches for the six PFC subregions in four association area fields and early auditory areas. The mean positions of the columnar patches in the cingulate, parietal, and temporal fields are shown in the right panel. (H) Visualization of topographic relationships in the cingulate and temporal fields. The line bars indicate the deviations of the true positions from the predicted ones. The injections shown on the left had at least one columnar patch in the projection field. (I) 3D medial and lateral views of the areas shown in (F)–(H). Colored areas in the frontal areas correspond to the similarly colored subregions in (H). OFC is hidden. See also Figures S4–S6.

A key to our project was the implementation of serial two-photon tomography (STPT)^28–30^ in the marmoset brain. STPT captures high-resolution serial section images in accurate 3D coordinates. Combined with enhanced GFP expression in a Tet-based adeno-associated virus (AAV) vector system, we achieved highly sensitive and detailed volume imaging of the entire brain, not available by conventional methods. This is illustrated by the reconstruction of columnar axonal spread for an exemplar dorsolateral PFC (dlPFC) injection (Figures 1A–1D: arrow and arrowheads 1–3; Videos S1 and S2). Depending on section obliquity relative to the radial axis, a single section may capture most of a projection column (Figure 1B) or only part of it (Figure 1A), but in all cases serial sections revealed the full 3D pattern (Figures 1C and 1D). Registration fidelity to the common template space^26^ was estimated to be 100–200 μm (Figure S1A; see STAR Methods), which enabled reliable automatic anatomical annotations (Figure S1B) and integration across multiple datasets. The slice interval was set at 50 μm, thereby enabling smooth conversion of cortical layers to a stack of flatmaps (“flatmap stack”) (Figure S1C) even in regions of cortical curvature (e.g., frontal pole, dorsomedial convexity) (see Figure S1E for distortion of intracortical areas). We separated the axon signals from background noise (e.g., lipofuscin granules; Figure S1F) using a machine learning-based algorithm, achieving more than five orders of magnitude range of signal intensity (Figure S2F). This wide intensity range is consistent with previous retrograde studies in macaques^31^ and marmosets.^32^ The distribution of the log_10_ values of projection intensity and its relationship to distance from the injection site along axonal trajectories was similar but not identical to that in previous studies (see Figure S2; also see STAR Methods). Pseudocolor scaling of logarithmic values revealed both the convergence of axons into patches and diffusely spread weak signals (Figures 1E–1G). The columnar nature of these exemplar patches is particularly evident in an oblique 3D view of PFC (Figure 1G). Importantly, the patches were not randomly scattered in the tangential domain, but are mostly grouped into rows, consistent with a stripe-like arrangement^13^ if the data were analyzed at lower resolution (see also Figure 2A).

Figures 1H and S1G show the estimated locations of injection sites on the cortical flatmap that includes a 117-area cortical parcellation (Figure S3) based on an architectonic analysis of a single hemisphere.^5,24^ The areal boundaries provide a useful reference frame, but our regional analysis was based on clustering the atlas areas into 6 larger PFC “core” subregions identified by geographic location, shaded in different pastel colors in Figure 1H and assigned geographic labels as indicated in the figure and legend. Figure 1I represents the overlay of projections from the six core PFC subregions using linear scaling. The projections from different subregions were largely segregated in the three posterior association area fields and had very different weightings, with dorsolateral PFC-dorsal (dlPFCd) projections (green) dominating the cingulate field, dorsolateral PFC-ventral (dlPFCv) projections (red) dominating the parietal field, and orbitofrontal cortex (OFC) and anterior cingulate cortex (ACC) projections dominating the temporal field. There is a quasi-orderly topographic representation in each of the association fields (see below and Figures S1H and S1I). Using this coverage, the distribution patterns and principles of patchy projections and diffuse spread are examined in detail below.

### Columnar projections recapitulate PFC topographic gradients locally in each association field

To characterize axonal convergence patterns in the cortex in greater detail, we searched for local maxima in the flatmap (Figure 2A) and identified 1,867 patches of strong labeling from all injections combined. To assess how consistently these patchy patterns are aligned as columns parallel to the radial axis, we binarized the tracer signals for morphological assessment. We found that more than half (54%, 1,008/1,867) of the binarized 3D clumps were solitary (disconnected from surrounding label) and were reasonably well approximated by ellipsoids. Of the 1,008 solitary patches, 569 (56%) had ellipsoids whose major axes were within 10° of the estimated radial axis (Figure 2B, green histogram and below the dotted line in scatter plot on right); and we consider all of these to be oriented radially. In the remaining 44%, most patches were insufficiently elongated to identify a clear axis of orientation, and most of them represent isolated patches in superficial layers (Figure 2B, red dots in scatter plot on right). For the 859 “connected” cases (46%), the averaged data suggested that most of them were associated with radially oriented signal convergence, especially in the upper layers (ULs) (Figure 2C). Indeed, a translucent display of UL portions of all patches aligned as a single stack (Figure 2D) indicated that patches were consistently narrow in diameter (~8–10 voxels) for both solitary (“Sol”) and connected (“Con”) cases. This corresponds to ~672–840 μm diameter in the flatmap stack (see Figure S1E). We conclude that the submillimeter connectivity patches, both local and long-distance (including those projecting outside PFC), predominantly have a radially oriented columnar architecture, although intensity profiles can vary considerably across layers, and many patches are restricted to superficial cortical layers. Accordingly, we refer to these as “columnar patches.”

Each injection generated a variable number of columnar patches within and outside the frontal cortex (Figure 2 E, one-way ANOVA, p < 0.05). Within the frontal cortex, subregions frontopolar cortex (FP), dlPFCd, and OFC injections averaged ~40–60 patches/injection, whereas dlPFCv, dorsomedial PFC (dmPFC), and ACC had significantly fewer. Outside the frontal cortex, dlPFCd, dlPFCv, and FP averaged ~10–20 patches/injection and the others had fewer still. A map of the total number of columnar patches (circle diameter) and the frontal/total ratio (circle hue) for each injection locus showed the total numbers highest for dorsolateral injectionsand high frontal ratiosmainlyin orbitomedial subregions (Figure 2F), suggesting regional differences in the number and distribution of columnar projections. Furthermore, the size of the solitary patches and the solitary ratio (solitary/all) differed across PFC subregions: in dlPFCv, dlPFCd, and OFC, the average patch size was relatively small, and the average solitary ratio was relatively high, whereas the converse was the case for dmPFC, FP, and ACC (Figures S4A–S4C). These observations suggest qualitative and quantitative differences in the interactions of each area with nearby and long-range neuronal populations.

Importantly, the distribution of columnar patches in cingulate, parietal, and temporal association cortices was systematically related to the location of injection sites (Figure 2G). In the cingulate and temporal cortex, the columnar patches from all six sub-regions reflect key aspects of PFC topography. Specifically, we could infer the mean target positions in each region based on the injection coordinates using polynomial regression models (Figures S4D and S4E). These models indicate that PFC topographic gradients are represented as shown in Figure 2H. The projections to the parietal cortex differed in that columnar patches arose only from dorsolateral injections (dlPFCd, dlPFCv, FP-red, green, cyan, Figure 2G). A seed analysis using all injections showed segregated projections from the anterior and posterior compartments of the dorsolateral frontal lobe, including premotor cortex—PM (Figure S4F). We also found columnar patches in early auditory areas (the core and belt) originating from two portions of dlPFCd and dlPFCv (Figure S4G). We propose that the patchy projections from the PFC take parallel pathways toward these extra-frontal fields, where they recapitulate key aspects of PFC topography in region-specific ways. Figures S4H and S4I show that these topographic projections are impressively similar to those reported for macaque lateral PFC.^25^

We also found orderly relationships in the distribution of columnar patches within the frontal cortex (Figures S5A–S5C). The frontal patches from each injection were distributed over a quasi-elliptical up to 1 cm tangentially. The mean shortest interpatch distance was ~1 mm (Figures S5D–S5G), similar to but modestly larger than reported in the macaque (500–600 μm).^13,16^ We also found that the pattern of projections to the contralateral hemisphere was strikingly similar to the ipsilateral projections, especially in the frontal cortex, as illustrated by comparing an ipsilateral to a mirror-flipped contralateral image that was adjusted for global intensity differences (Figure S5H). Although the overlay of the contra- and ipsilateral projections was often slightly offset, comparison with shuffled data indicates a largely symmetric pattern of bilateral projections (Figures S5I–S5K). Patchy projections appear to be much less prominent in the mouse PFC (Figure S6), suggesting they may have a distinctive role in the primate brain.

### Diffuse projections recapitulate PFC topographic gradients globally and locally

**Figure 3.**
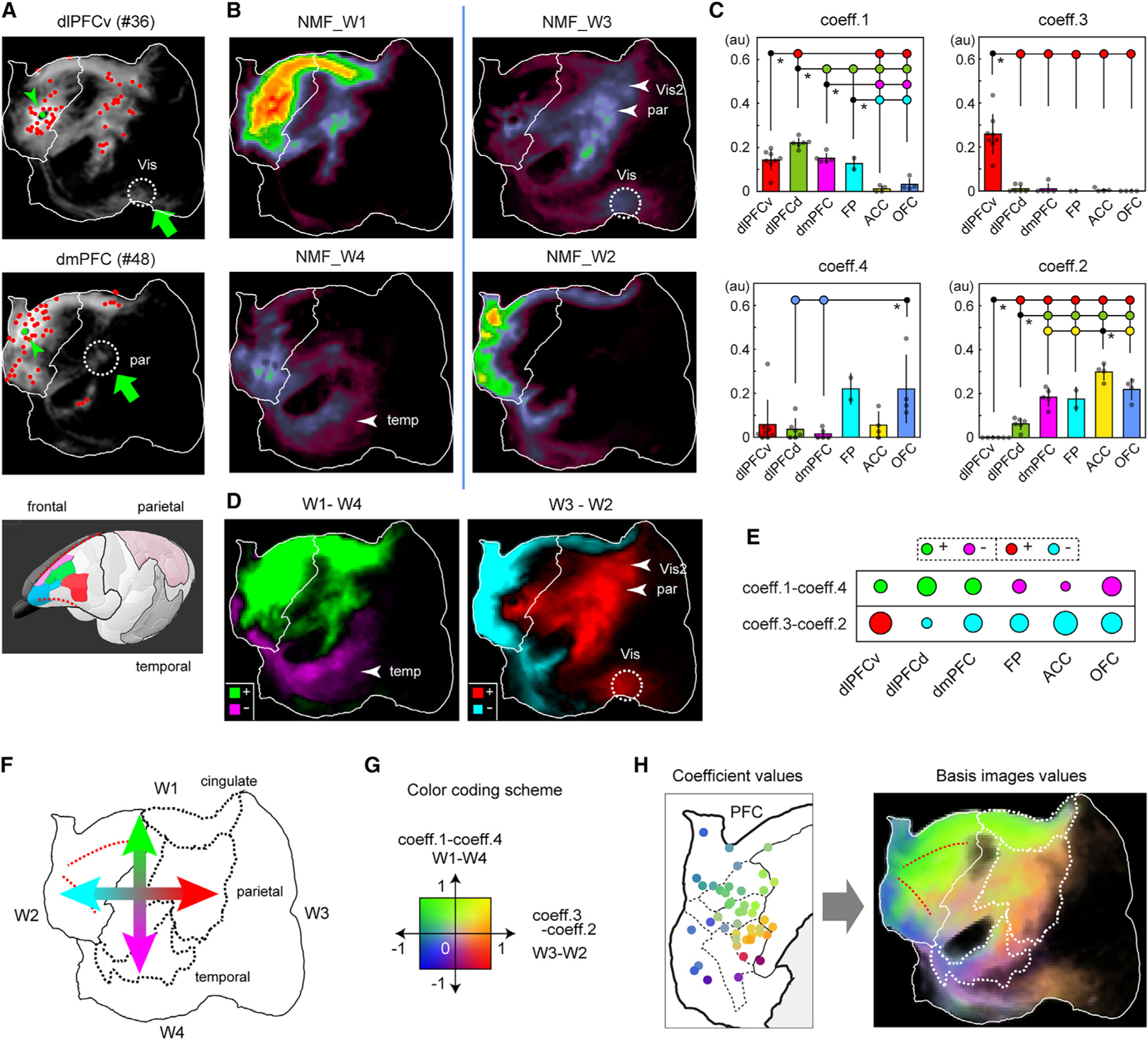
Characterization of diffuse corticocortical projections in log-scale by nonnegative matrix factorization (NMF) analysis (A) Log-transformed tracer images for two injection cases. The red dots indicate columnar patches detected in these cases. Note the presence of weak signals in the dotted circles indicated by green arrows, where we detected no columnar patches. par, parietal area; Vis, visual area. The bottom panel (same as Figure 2I) shows the borders of the frontal, parietal, and temporal areas. (B) Four components (basis images) obtained by the NMF analyses. The intensity is represented in pseudocolor (Videen palette). (C) Coefficients of each injection, grouped by PFC subregions. Error bars indicate SD. See the legend in Figure 2E for statistical significance. (D) The subtracted values for two pairs of NMF components (NMF_W1-W4 and NMF_W3-W2) are shown with different colors for the positive and negative values (see STAR Methods). (E) Representation of the subtracted values of the coefficients. The averaged values after subtraction are shown as circle areas with different colors for the positive and negative values. (F) Schematic representation of the orthogonal gradients observed in (D). Frontal, parietal, and temporal areas are as shown in the lateral 3D view above. (G) 2D color map used to represent the variable combinations of the subtracted values for the components and coefficients. (H) Display of the ratio of the components at different cortical locations (right) and coefficients for each injection (left) using the 2D color map in (G). These colors were assigned to regions above the threshold value. Below-threshold regions with almost no axon signals (e.g., early somatomotor, insular, and visual areas) are shown in black. Note that these colors are not the same as shown in (D) or (E). See also Figures S7–S9.

Figure 3A shows a highly distributed pattern of weak anterograde labeling visible using log scaling and gray-scale encoding for injections in dlPFCv (top) and dmPFC (middle)—Figures S7 and S8A show additional injections. Diffuse projections were even observed in regions lacking any columnar patches (Figure 3A, green arrows), suggesting their regional specificities are not identical. To analyze the spatial patterns of the diffuse projections, we performed a nonnegative matrix factorization (NMF) analysis. Figures 3B and 3C show the results of NMF, which generated four common components (basis images) (NMF_W1, 2, 3, and 4) and associated coefficients (coeff. 1, 2, 3, and 4). The reconstruction by these values was in general well correlated with the original image (average correlation = 0.93) (Figure S8B). The four basis images can be grouped into two quasi-orthogonal pairs on the flatmap (Figure 3B; separated by a vertical blue bar). A dorso-medial pair (NMF_W1) vs. ventro-lateral (NMF_W4) pair is reminiscent of the dual origin concept proposed by Pandya and coworkers.^7^ An anterior/rostral (NMF_W2) vs. posterior/caudal (NMF_W3) pair resembles the antagonism between the “apex transmodal network” vs. the “canonical sensory-motor network” (primarily visuo-motor and distinct from the somato-motor network) previously described in the marmoset^33^ based on retrograde tracer data.^6^ The subregion-specific patterns are reflected in the coefficient values for these components, namely, coeff. 1 through 4 (Figure 3C, one-way ANOVA, posthoc Tukey test, *p < 0.05). Notably, dlPFCv was the only subregion with high coeff. 3 values, indicating that NMF_W3 was nearly unique to dlPFCv. In other cases, 2, 3, or 4 subregions had substantial coefficients, suggesting that the positional gradients of injections strongly influence these coefficients. To visualize the differential contributions of the four parameters in a simpler form, we combined the antagonistic pairs by subtraction, retaining most of the original information in the positive and negative domains (Figure 3D). Similarly, we combined the coefficients by subtraction (Figure 3E). This data conversion highlighted the orthogonal trends of NMF_W1 through W4 (Figure 3F). Using a 2D color index strategy^25^ (Figure 3G), these trends were represented in a single map (Figure 3H, right panel). A similar strategy converted the coefficient values of each injection by hue representation (Figure 3H, left panel). The gradual progression of hues suggests graded changes in the combination of coefficient values according to injection location. Indeed, we were able to model their relationship by polynomial regression (Figure S8C, right two panels). Since the projection patterns reflect the coefficient values, we infer that the source region mainly projected to target regions similar in hues (compare the left and right panels of Figure 3H).

Given the presence of global gradients for the diffuse projections, we quantified the patch intensity and patch numbers and found that the patchy projections also show similar gradients (Figures S8D and S8E). Conversely, when we examined the center of mass of diffuse projections, it showed a modest but genuine bias (Figure S9A), albeit not as pronounced as for the patchy projections. Furthermore, NMF analyses using only the local values successfully visualized the recapitulation of the PFC layouts in the cingulate and temporal areas, although some variability was observed in the distribution of the coefficient values (Figures S9B–S9G). We suggest that PFC gradients are mapped globally across the cortical hemisphere to determine the overall projection patterns and are also mapped locally in each association field to determine the positioning of both patchy and diffuse projections.

### Columnar and diffuse projections have different laminar profiles

**Figure 4.**
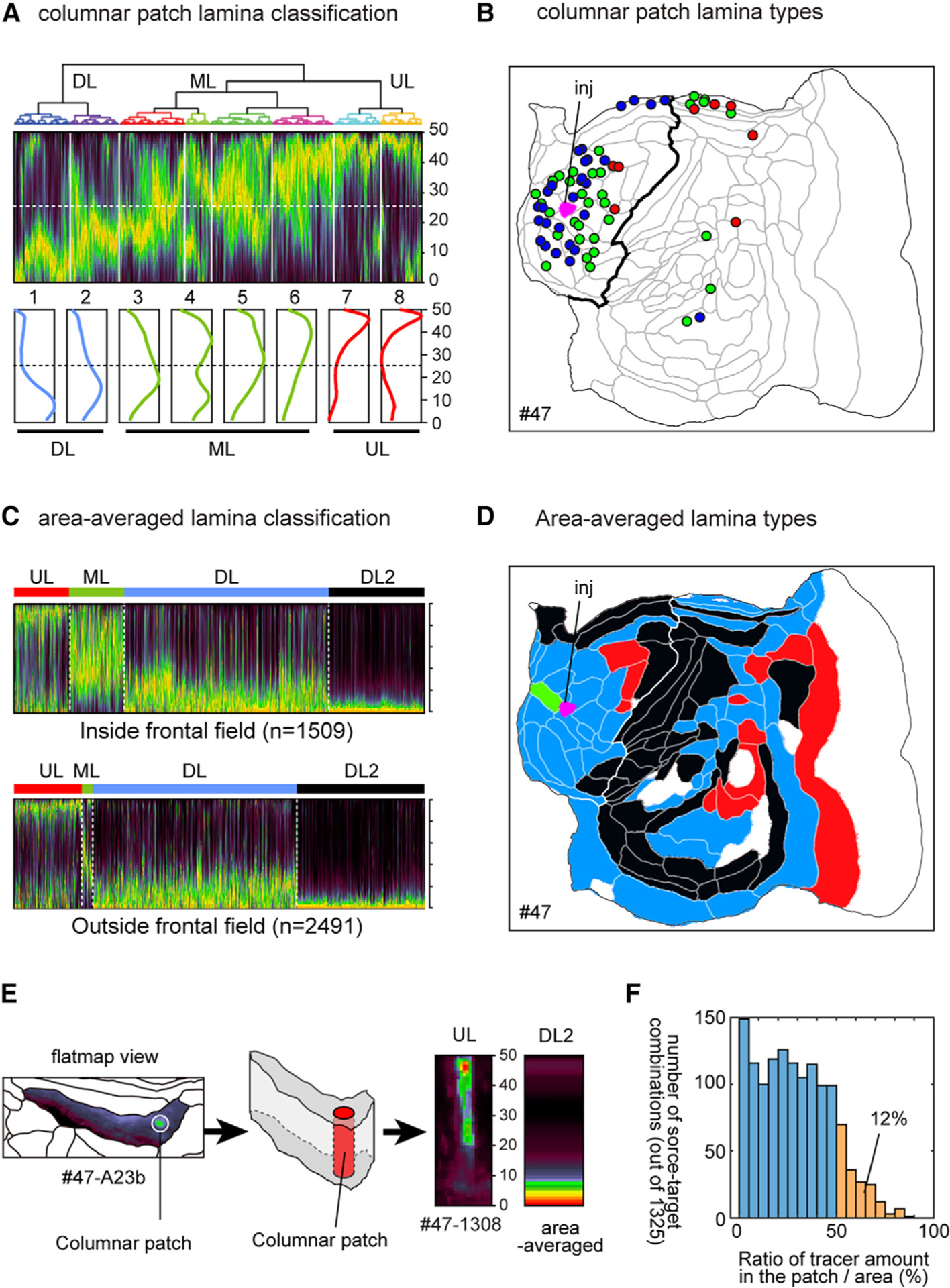
Differential laminar preferences of the columnar patches and diffuse projections (A) Classification of the laminar patterns of the columnar patches. Laminar profiles for the 1,867 columnar patches were arranged based on the hierarchical clustering. The averaged profiles of eight clusters are shown below and are further classified into the upper layer (UL), middle layer (ML), and deeper layer (DL) groups. (B) An exemplar map showing the laminar types (red for UL, green for ML, and blue for DL) of columnar patches for sample #47. The thick line indicates the frontal cortex border. (C) Classification of the laminar patterns of the area-wise connectivities. Area-based lamina profiling (integrating patchy and diffuse projections within each area) of 4,000 source-target combinations into four groups. (D) An exemplar map showing the area-averaged laminar types (red for UL, green for ML, blue for DL, and black for DL2) for sample #47. (E) A schematic diagram explaining the differential lamina patterns for the columnar patches and area averages. An example patch (ID1308 of sample #47) in area A23b is shown. (F) A histogram showing the ratio of the amounts of tracer within columnar patches vs. that for the entire area (diffuse + patchy). Those over 50% (shown in orange) constituted only 12% of the 1,325 source-target combinations having columnar patches. See also Figure S10.

The laminar profile of axon terminals has been used to infer hierarchical relationships between connected areas.^34–37^ To investigate this issue in our data, we classified the laminar patterns of columnar patches by hierarchical clustering (Figures 4A and S10A). Based on the stereotypical patterns reported previously, particularly for the visual cortex, one might suspect that clusters targeting upper and deeper layers (grouped as ULs and DLs, respectively) represent feedback connections, whereas those that target widely across the middle layers (MLs) might be feedforward or lateral (horizontal) connections. We obtained eight clusters that were combined into DL, ML, and UL groups, with a majority being ML type (green), and examined their areal distribution patterns (Figure 4B for #47; Figure S10B). These examples showed a tendency for similar laminar types to cluster, albeit with considerable intermingling. Next, we examined the laminar profiles of the diffuse projections. As regions of interest (ROIs) for profiling the continuous diffuse patterns, we used the 117-area parcellation (Figure S3A). We selected 4,000 injection-to-target area pairs out of 44 × 117 possible combinations (78%) as putatively positive (see STAR Methods) and classified them by hierarchical clustering into four types: UL, ML, DL, and DL2 (Figure 4C). The incidence of ML laminar types for diffuse terminations was much lower than for columnar patches in the frontal cortex (Figure 4C, upper panel) and even more so outside the frontal cortex (Figure 4C, lower panel), as is also apparent for the exemplar injection (#47, Figure 4D). In many areas, the area-wise laminar types differed from the dominant patch types, presumably because strong but restricted columnar signals in the MLs were dwarfed by diffuse, widespread projections in DLs (Figures 4E and 4F). Figure S10F compares the percentage of the dominant laminar types of the columnar patches with that of the area-wise measurement (left panel), which showed a decrease of the ML type and an increase in DL and DL2 types. Importantly, some areas are reciprocally connected with one another by DL pathways in both directions (Figure S10G). If these represent genuine inter-areal connections and not just fibers of passage, this pattern would be inconsistent with a traditional hierarchical model in which DL represents a feedback pathway (see discussion).

### Reciprocity of corticocortical projections determined by anterograde/retrograde double-tracing

**Figure 5.**
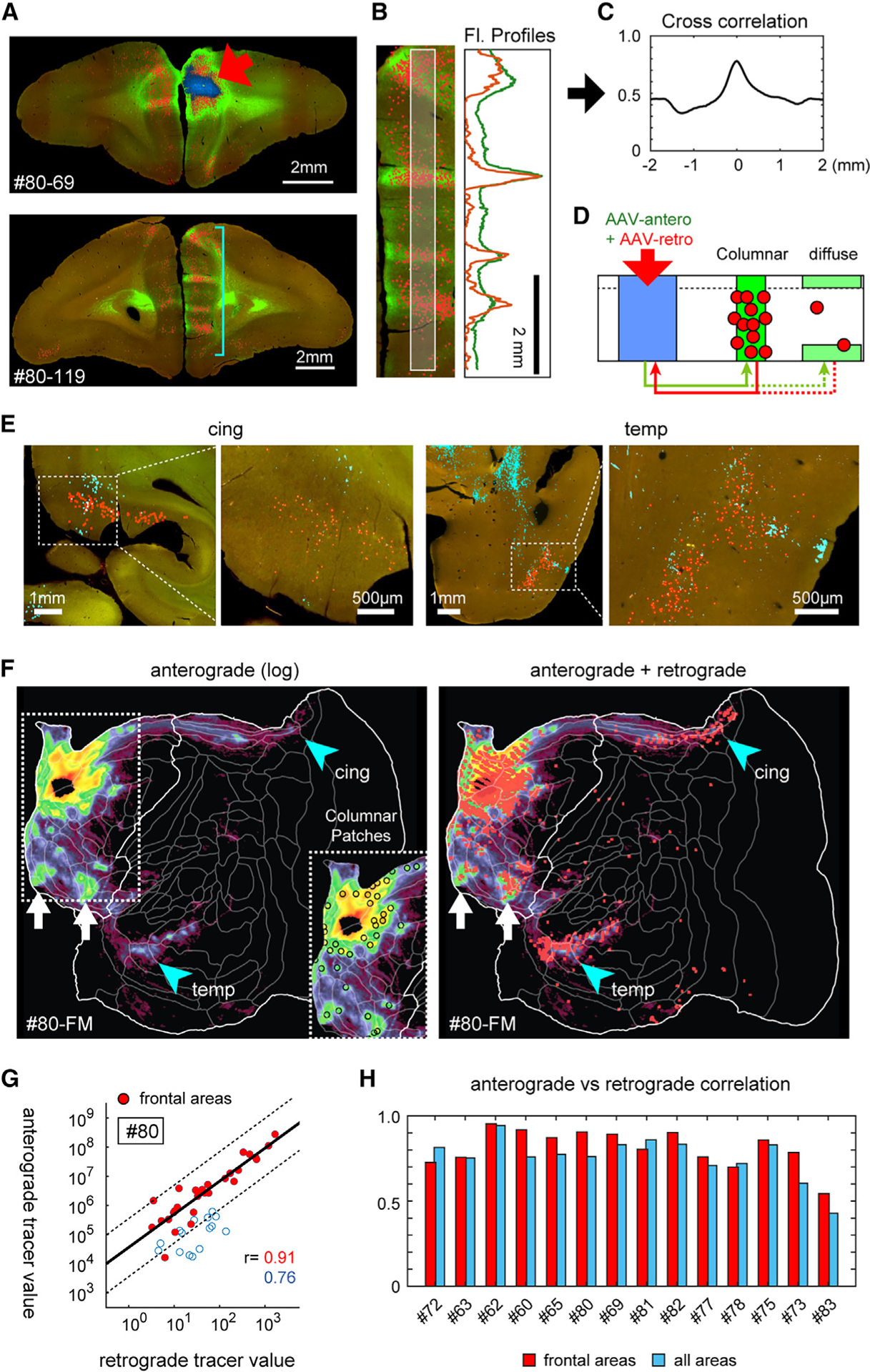
Reciprocal connectivity of corticocortical projections (A) Example images showing colocalization of anterograde and retrograde tracers. The injection site is indicated by the blue overlay and a red arrow. Red dots: retrogradely labeled cell nuclei. (B) The fluorescence intensities in the red and green channels were measured along the vertical strip in the middle layers (outlined by white lines) to show colocalization. (C) Cross-correlation analysis to measure the coincidence of the anterograde and retrograde peaks. (D) A schematic representation of reciprocal columnar connectivity visualized by co-injection of anterograde and retrograde AAV tracers. (E) Colocalization of the anterograde (cyan) and retrograde (red) signals (segmented) in the cingulate and temporal regions. (F) Left: anterograde label alone (pseudocolored in log-scale). Inset shows the positions of the columnar patches. Right: retrogradely labeled neurons (red dots) overlaid on the anterograde data. Because we stained only one in ten sections for the retrograde tracers, there were gaps in the flatmap (e.g., around the injection site). (G) Area-based comparison of anterograde and retrograde signals displayed on a log-log-scale. The solid line indicates the best fit for the frontal areas (red dots) and the dashed lines above and below indicate 10-fold differences. The open circles indicate areas outside the frontal field. The correlation coefficients (r) for the frontal areas only and for all areas combined are shown in red and blue lettering, respectively. (H) Correlation coefficients of the retrograde and anterograde signals in the frontal areas only (red bars) and for all areas (blue bars). Two injections that intruded into the white matter were excluded from the analysis. The 5 cases with noisy retrograde signals (#77–#83 on the right) tended to show lower correlations (see Figure S11). See also Figure S11.

It is well established that most but not all inter-areal corticocortical connections are reciprocal,^8,15^ but the degree of spatial precision largely remains to be determined. Thus, we investigated whether both patchy and diffuse projections in the marmoset PFC are associated with reciprocal projections. To test this, we co-injected a non-fluorescent retrograde tracer in 14 cases and compared its distribution pattern with the anterograde tracer by immunostaining. Figure 5 shows our main findings using case #80 as an exemplar. The retrograde signals exhibited striking colocalization with the anterograde signals by visual inspection (Figure 5A), in densitometry (Figure 5B) and cross-correlation analyses (Figure 5C). Retrogradely labeled neurons occurred in all cortical layers except layer 1 in many patches (Figures 5A and 5B) but could be concentrated in DLs outside patches (Figure 5E). Strong anterograde columnar patches were consistently associated with moderate to strong retrograde labeling. We also observed a widespread but sparser pattern of retrogradely labeled neurons in locations containing diffuse anterograde label (Figure 5E). Thus, reciprocal connectivity applies to both patchy and diffuse projections (Figure 5D).

To quantify these relationships, we projected the retrograde data into a flatmap for comparison with the anterograde data (Figure 5F). We confirmed that strong anterograde signals were consistently associated with clusters of retrograde signals (e.g., Figure 5F, white arrows, compare both panels). We also observed the colocalization of retrograde signals with the sparse diffuse anterograde signals in the temporal and cingulate cortex (cyan arrowheads). To quantify this reciprocity, we integrated the total amount of anterograde and retrograde tracer signals in each cortical area and plotted their correlations (Figure 5G). The correlation was high (r = 0.94) in the frontal cortex (red dots), where strong columnar patches were abundant, and lower but still robust (r = 0.76) when non-frontal areas (open circles) were included, where diffuse signals dominated. Similar results were found in the other 13 dual-injection cases, supporting the generality of these observations (Figures 5H and S11). Furthermore, we also found a generally strong correlation of area-wise anterograde/retrograde labeling for the contralateral projections (Figures S5L and S5M).

### Corticostriatal projections also consist of patchy and diffuse projections

**Figure 6.**
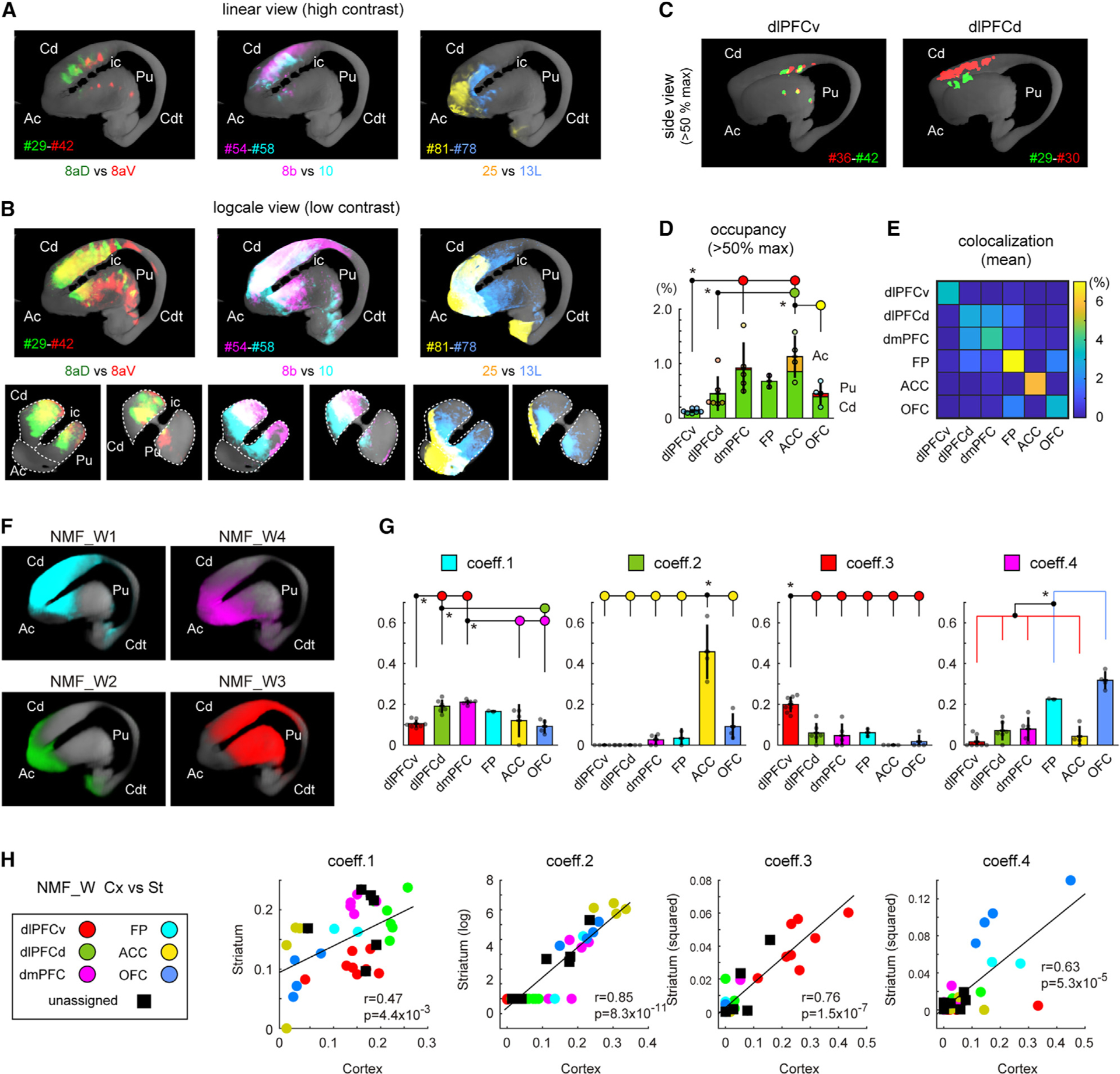
Characterization of patchy and diffuse corticostriatal projections (A) Three-dimensional (3D) representation of corticostriatal projections for three pairs of injections, shown with linear scaling, showing patchy axonal convergence. See Video S3 for bilateral rotation views. Ac, nucleus accumbens; Cd, caudate nucleus; Cdt, the tail of the caudate nucleus; Pu, putamen; ic, internal capsule. (B) The log-scale view of the same data shows a diffuse spread of the tracer signals. Note that the contrasts of the intensity differences are high and low for the linear and log-scale views, respectively. The lower panels show coronal section views at two AP positions. (C) Interdigitation of patchy projections visualized by binarization at 50% of the maximum values. (D) Occupancies of the binarized signals in the total striatal volume. The averaged occupancies in the caudate, putamen, and nucleus accumbens are overlaid. (E) Averaged colocalization ratio within and across subregions. See also Figure S12C. (F) NMF_W1 through W4 in 3D representation. (G) Coefficients of different subregions for each component. Data are represented as mean ± SD. (H) Similarity of coeff. 1 through 4 between cortical and striatal projections shown by scatter plots. The values for striatal coefficients in the scatter plots show the original (for coeff. 1), log-transformed (for coeff. 2), or squared (for coeff. 3 and 4) values, which are correlated with the non-modified cortical coefficient values (see Spearman’s rank correlations and p values in each panel). See also Figure S12.

PFC has massive unidirectional projections to the striatum, constituting the first step of the cortico-basal ganglia-thalamo-cortical loop that plays an integrative role in goal-directed behaviors.^38,39^ Anterograde tracer injections in the macaque showed that the corticostriatal projections include both focal and diffuse projections,^40^ which may correspond to patchy and diffuse projections in our study. In marmosets, we also observed dense patches of anterograde label surrounded by sparser and more widespread anterograde label; here, we characterize these patterns in detail using an analysis strategy similar to that applied above to corticocortical projections. The striatum includes the caudate nucleus (Cd), putamen (Pu), nucleus accumbens (Ac), and tail of the caudate nucleus (Cdt), which are implicated in different functional circuits.^39^ Typical termination patterns for three exemplar pairs of injections in these structures are shown using linear scaling (Figure 6A) to emphasize the strong patchy projections, and in log-scale view (Figure 6B) to emphasize the diffuse spread of weaker projections. In the linear view, we observed multiple patches along the rostrocaudal axis mainly within the Cd, consistent with previous observations in macaques.^41^ The patches were discrete for dlPFCd (#29, green) and dlPFCv (#42, red) injections (Figure 6A, left panel) and were more distributed for other injections, particularly for the A25 injection (#81, yellow) that primarily targeted the Ac (Figure 6A; see also Video S3). In the log-scale view, the tracer spread widely and there was extensive overlap across the different injections (Figure 6B, lower panels). The patchiness of corticostriatal projections was evaluated by measuring the signal spread at a threshold of half the maximum value (Figures 6C and 6D). Strong signals were concentrated in the Cd, except for the A25 injection (targeting Ac) (Figure 6D). The projections from dlPFCv were the most focal among the subregions, whereas those of dmPFC and ACC projections were the broadest, occupying more than 7-fold more space. The location of these projections varied systematically within the Cd, with little overlap between adjacent subregions (Figures 6E and S12C). As with the corticocortical projections, we could predict the centers of patches based on the injection coordinates using polynomial regression models (Figures S12D–S12G). These observations support the importance of topography in determining the specificity of patchy corticostriatal projections, consistent with previous studies in the macaque.^40,42^ The axonal terminations included fine axonal fibers with bouton-like varicosities (Figures S12A and S12B).

To characterize the distribution of diffuse projections, we performed NMF analyses and generated four components (Figure 6F). The NMF_W1 component, spanning most of Cd plus the anterior Pu, was strongly represented in all PFC subregions (Figure 6G, coeff. 1), suggesting that the Cd is an important target for all the PFC subregions. Other striatal regions (e.g., Pu, Ac, and Cdt) also received projections from some subregions of the PFC (compare Figures 6F and 6G). Interestingly, the profiles of these coefficient value sets were similar to those of the corticocortical projections (Figure 6H; Spearman’s rank correlation test). These observations strongly suggest that the global patterns of corticocortical and corticostriatal projections are governed by similar PFC topographic gradients, albeit in a nonlinearly skewed form. Visualization by the color indexing strategy confirmed that the PFC gradients were recapitulated globally in the striatum (Figure S12H).

## DISCUSSION

### Topographic gradients in the primate brain

**Figure 7.**
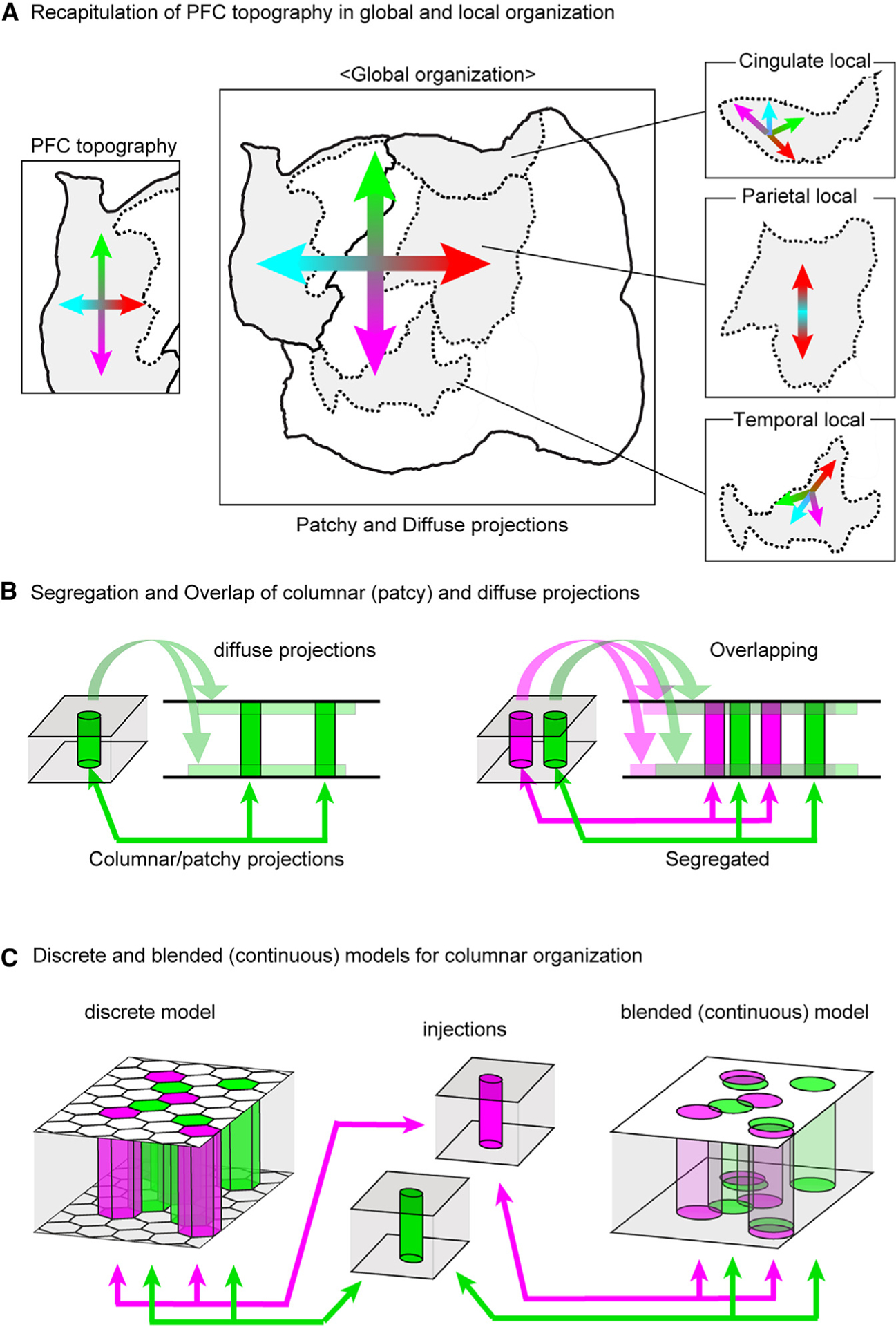
Summary of differential areal and lamina patterns of the columnar patches and diffuse projections (A) Schematic representation to show recapitulation of the PFC gradients in the global pattern of the diffuse projections and the local patterns of the columnar patch distributions. The cingulate and temporal fields each receive columnar projections from all six PFC subregions, whereas the parietal field receives columnar projections only from the dorsolateral surface. Although skewed, the layout in each field resembles the layout in the PFC. (B) Differential lamina preferences and overlap of columnar and diffuse projections. Columnar projections extend mainly along the radial axis and converge on small patches, whereas the diffuse projections spread widely within the superficial and deep layers. Because of such differential spread, the columnar projections are expected to segregate even from the nearby regions (right, green and magenta), whereas the diffuse projections show extensive overlap in the target regions (mixed color). (C) Discrete vs. blended (continuous) models for columnar organization. The discrete model postulates columnar modules with well-defined borders and no overlapping inputs and outputs with neighboring columns, whereas the blended (continuous model) postulates no such borders. If the former model is correct, any two remote injections would always show complete overlap or complete segregation. Otherwise, partial overlap (blending) would be a common occurrence.

We found that both patchy and diffuse projections recapitulate PFC topography in their local and global patterns (Figure 7A). Whereas the patchy projections showed segregated parallel streams to extra-frontal association areas, the diffuse projections spread widely and overlapped extensively. PFC projections are considered a mixture of these projection patterns (Figures 7A and 7B). Topographic organization has been proposed as a key organizing principle for various PFC extrinsic projections^34,39,41,43–46^ and functional properties.^47–49^ A recent study of electrical microstimulation (EM)-fMRI reported a topographic mapping of lateral PFC functional connectivity in five association cortex regions.^25^ Our datasuggest thata similar topographic mapping of anterograde connectivity occurs in at least three cortical regions in the marmoset. Furthermore, it likely involves orbitomedial PFC and applies to corticostriatal connections as well. Precise correspondences between macaque and marmoset association areas have yet to be fully established, despite many cytoarchitectonic and connectional similarities.^5,24^ The presence of gradients that are similar but not identical in the temporal, parietal, and cingulate fields provides important insights into both conserved and divergent connectional architecture across primate species. Macroscale network organization in humans may share features in common with marmosets,^33,50^ suggesting the generality of dual global/local topographic organization across the primate line-age. Although we consider our evidence for topographic mapping to be compelling, we observed considerable diversity in patch distributions (e.g., Figures S5A–S5C). Considering the variability even in the same individual in EM-fMRI mapping,^25^ mesoscale connectivity may include a stochastic component. Alternatively, individual differences that remained after registration may account for our variabilities.

### Functional implications of PFC connectivity patterns

The concept of “parallel distributed networks” has been used to characterize subdivision-specific connectivity patterns in the macaque PFC^45^ as well as parallel interdigitated networks in fMRI studies of individual human subjects.^51^ We consider this concept applicable to the patchy projections in our study (Figure 7B, right panel, green and magenta columns). An intriguing question is whether each column in PFC is a discrete entity that has well-defined borders with minimal overlapping inputs and outputs with neighboring columns (Figure 7C, left). Alternatively, a column identified by one tracer injection might partially overlap and be “blended” with a column identified by a tracer injected in a different location (Figure 7C, right). Our observation that anterograde and retrograde columnar patches were in most cases precisely coextensive (Figures 5A–5C) is consistent with the discrete columnar model but does not prove it. If further experiments indeed confirm the discrete columnar model for PFC, a host of fascinating questions arise: how many columns are in each PFC area? How many other columns does a given PFC column project to and receive inputs from? Are there gaps between neighboring columns, or is the PFC completely tiled by a mosaic of columns? How do neighboring columns differ in connectivity and function, gene expression patterns, and/or cell type composition? Recent advances in anatomical tracer methods,^52,53^ submillimeter fMRI in monkeys^54^ and in humans,^55,56^ and spatial transcriptomics^57^ will likely yield important insights regarding these issues.

A discrete columnar system in PFC would differ fundamentally from columnar systems in the visual cortex that have been intensively studied, particularly in the macaque (see introduction). An iso-orientation domain in V1 is in essence a thin ribbon that winds through the cortical sheet, but the ribbon lacks a well-defined thickness because orientation preferences change continuously rather than in discrete steps. An ocular dominance stripe in V1 has a finite thickness (width) related to eye-specific geniculocortical terminations, but along the length of a stripe, features such as orientation and spatial frequency appear to be mapped continuously.^10,58^ Area V2 has a tripartite arrangement of thick stripes, thin stripes, and interstripes and putative columnar systems for representing multiple dimensions, including orientation, binocular disparity, and hue (reviewed in Vanni et al.^9^ and Sincich and Horton^59^). In higher visual areas, modular organization has been reported in area V4 related to color and shape and in the inferotemporal cortex related to faces, bodies, color, disparity, and objects.^11,60–62^ However, we are not aware of compelling evidence in any extrastriate visual area for discrete columns at a submillimeter scale of the type hypothesized for PFC above and previously.^12–16^ On the other hand, columnar patches found in premotor, cingulate, parietal, and temporal areas in our study and in macaque anterograde studies^63^ may well reflect a similar columnar system. Multi-electrode recordings in macaque lateral PFC suggest a spatially non-monotonic tangential correlation structure^64^ to which patchy projections might contribute.

In contrast to the patchy PFC columnar system, diffuse projections spread widely and overlap extensively (Figure 7B, mixed color area in the top and bottom layers). An important question is the degree to which anterograde signals in DLs represent fibers of passage rather than just axonal terminations. In this regard, our confocal microscopy observations suggest the presence of boutons along axon fibers in DLs (Figure S8F). The dual tracer experiments involving DL and DL2 domains suggest a modest but non-zero incidence of reciprocal connections (Figure S11C). Thus, while we acknowledge that fibers of passage may be common, diffuse projections appear likely to contribute functionally relevant connections. Such connections might have significant modulatory effects when a large population of neurons exhibit coordinated activity. Resting-state fMRI in marmosets suggests the existence of such coactive networks involving frontal areas, including a candidate for the default mode network.^17,20^ This raises the intriguing question of the degree to which diffuse vs. patchy projections arise from and/or terminate on separate vs. overlapping neuronal populations.

Neural recordings from the monkey PFC suggest that computations in the PFC emerge from the concerted dynamics of large populations of neurons,^65^ in which multidimensional activities may be superimposed.^66,67^ The anatomical features we found for PFC might contribute to the segregation and integration of population activities. For example, the reciprocal columnar architecture is well-suited for forming recurrent networks. As discussed above, neural computations might occur in completely or partially segregated parallel networks with modulation through diffuse connectivity (Figure 7C). It is also important to know how lamina-specific connections contribute to the network organization. We find it notable that the laminar profile of projections commonly differs for the patchy and diffuse projections (Figure 7B). Laminar profiles have been considered to reflect the directionality of information flow and hence the hierarchical organization of cortical areas.^8,35,37^ Our data suggest inconsistencies with a traditional hierarchical scheme for PFC organization, but the issues of laminar profiles and hierarchical organization will benefit from further analyses using more extensive datasets, which are currently ongoing. Progress on this front may provide insights as to how layer-specific cell types contribute to the formation of PFC circuits.^13,16^ Another fundamental issue is the need for a more accurate cortical parcellation of marmoset PFC, incorporating multimodal data including gene expression data. Finally, we note that our marmoset PFC connectivity database offers many exciting opportunities to perform fine-grained analyses that were not previously possible and should contribute to our understanding of PFC structure and function in primates.

## STAR★METHODS

### RESOURCE AVAILABILITY

#### Lead contact

Further information and requests for resources and reagents should be directed to and will be fulfilled by the lead contact, Akiya Watakabe (akiya.watakabe@riken.jp).

#### Materials availability

Plasmids generated in this study have been deposited to Addgene: pAAVTRE3_Clover (#135179), pAAV-TRE3_ mTFP1-Vamp2 (# 201214) and pAAV-EF1_Cre (#201198).

#### Data and code availability

All the section image data from STPT, standardized 3D data for the whole brain, and the flatmap stack data for the cortical signals of the marmosets have been deposited at Brain/MINDS data portal (https://dataportal.brainminds.jp/marmoset-tracer-injection) and are publicly available as of the date of publication. The high-resolution images used in the paper are labeled with the marmoset number and the section number (e.g., #82–139) and are available through the zoomable section viewer. In addition to the visualization and search tools available at these sites, users can download standardized 3D data for tracer segmentation, fluorescence-weighted segmentation, AAV-transduced cell segmentation, and original and standardized flatmap stack data in nifti format and other information from The Brain/MINDS Marmoset Connectivity Atlas^26^ (https://dataportal.brainminds.jp/marmoset-connectivity-atlas) or on request. The mouse analyses used the existing, publicly available data of the Mouse Brain Connectivity Atlas^28,29^ (https://connectivity.brain-map.org/). The accession numbers for the used datasets are listed below. Other microscopy data reported in this paper will be shared by the lead contact upon request.

Any additional information required to reanalyze the data reported in this paper is available from the corresponding authors upon request.

### EXPERIMENTAL MODEL AND SUBJECT DETAILS

All experimental procedures were carried out following the National Institute of Health Guide for the Care and Use of Laboratory Animals (NIH Publications No. 80–23) revised in 1996 and the Japanese Physiological Society’s “Guiding Principles for the Care and Use of Animals in the Field of Physiological Science,” and were approved by the Experimental Animal Committee of RIKEN (W2020–2-009(2)). The age and sex of the marmosets used are listed in Table S1. We did not distinguish these factors in this study.

### METHOD DETAILS

#### Marmoset experiments

In our standard procedure, we acquired a structural MRI scan (T1w, T2w, DWI) using a 9.4-T BioSpec 94/30 unit (Bruker Optik GmbH, Ettlingen, Germany) and a transmit and receive coil with an 86-mm inner diameter^76^ under anesthesia at least one week before surgery to plan injections. The presumed positions of cortical areas were determined by registration of the Brain/MINDS reference^24^ and the stereotaxic positions were aligned by using the interaural plane and the anterior limit of the cortex. Surgery for tracer injections was performed as previously described with some modifications.^77^ Pressure injection was performed using a glass micropipette with an outer diameter of 25–30 μm connected to a nanoliter 2000 injector with a Micro4 controller (World Precision Instruments). For exposed cortical areas, we injected 0.1 μl each of tracers at two depths (0.8 and 1.2 mm from the surface), aiming to deliver the AAV to all cortical layers. For deep injections (e.g., OFC), we injected 0.2 μl of tracer at one depth. With these volumes, we did not experience overflow, unlike the experience in our previous study.^77^ To avoid fluorescence cross-talk, all subjects received a fluorescent tracer at only one site. However, in some cases, we injected nonfluorescent tracers, such as BDA, into several other locations, but these results are not reported here. After surgery, the marmosets were returned to the cages and euthanized four weeks later.

The variability of injections (injection volume and depth) was analyzed in a post-hoc manner based on the spread of AAV-transduced cell bodies (see “image processing pipeline” below).

#### AAV tracers

We used a TET system to amplify fluorescence signals.^68,69,78,79^ This system labeled all the known projections from the PFC and did not show strong cell-type bias. The detection of two types of projections is also not due to our TET labeling system, as we observed a similar convergence and spreading of axon fibers using the conventional biotinylated dextran amine (BDA) method (data not shown). Our standard tracer mix included AAV1-Thy1S-tTA (1 × 10e9 vg/μL), AAV1-TRE-clover (1 × 10e9 vg/μL; a GFP derivative), and AAV1-TRE3-mTFP1-Vamp2 (0.25 × 10e9 vg/μL; cyan fluorescent protein targeted to the pre-synapse). In later experiments, we also included AAV2retro EF1-Cre (1.5 × 10e9 vg/μL) in the tracer mix for co-injection. We deposited plasmids for these AAV vectors to Addgene (https://www.addgene.org/).

#### PFC injections

In this study, we present the data from 44 high-quality datasets involving injections into various frontal areas of the left hemisphere (Figures 1H and S1G; Table S1). To plan injections, we referred to previous studies.^5,6,46,80,81^ We divided the frontal areas of marmoset into nine subregions, including the frontopolar cortex (FP), dorsolateral PFC_ventral (dlPFCv), dorsolateral PFC_dorsal (dlPFCd), dorsomedial PFC (dmPFC), ventrolateral PFC (vlPFC), anterior cingulate cortex (ACC), and orbitofrontal cortex (OFC), plus premotor areas (PM) and dorsal ACC (dACC) (Figure S1G). The ACC subregion in this study comprises areas A32, A14, A25, and A24a and corresponds to the subgenual anterior cingulate cortex (sgACC) and perigenual ACC (pgACC) in other studies^82^ and was differentiated from the dACC comprising A24b and A24c. We injected most densely into the aforementioned dlPFC subdivisions dlPFCv and dlPFCd, corresponding to areas 8aV/A45 and 8aD/46D, respectively, by our cortical annotation; dlPFCv most likely overlaps extensively with the frontal eye field (FEF).^33,83^ The relationship of “dlPFCd” to macaque areas 46, 9/46, and 8aD is currently unclear. However, our results suggest the inclusion of A46-like areas in dlPFCd, judging from the auditory projection (Figure S4G). Throughout the manuscript, we used 29 datasets with clean injections in six PFC subregions (FP, dmPFC, dlPFCd, dlPFCv, ACC, and OFC) for subregion comparison. These injections are shown color-coded in Figures 1H and S1G. Some of the injections localized at the border of these subregions and were used only for parcellation-free analyses (gray dots in Figure 1H). Injections into the vlPFC, PM, and dACC subregions were also used only for the selected analyses.

#### Serial two-photon tomography imaging (STPT)

After transcardial perfusion with 4% paraformaldehyde in 0.1 M phosphate buffer (pH 7.4), the marmoset brains were removed, post-fixed at 4 °C for 2–3 days, and transferred to 50 mM phosphate buffer (pH 7.4). All marmoset brains had an ex vivo MRI scan (T2w and DWI) before further processing. MRI was performed using a 9.4-T BioSpec 94/30 unit (Bruker Optik GmbH) and a transmit and receive solenoid type coil with a 28-mm inner diameter.^76^ Agarose embedding was performed as previously described^30^ in 5 % agarose using a custom-made mold. Before embedding, we treated the brain with 1 mg/ml collagenase (Wako 031–17601) at 37 °C for one hour and manually removed the meninges as thoroughly as possible. This step ensured direct crosslinking of agarose to the brain for stable sectioning at a 50-μm interval. STPT was performed as described^28–30^ using TissueCyte1000 or TissueCyte1100 (TissueVision). We immersed the agarose block in 50 mM PB supplemented with 0.02 % sodium azide for imaging and sectioning. We used a 920–940 nm laser for excitation, and the two-photon excited fluorescence was recorded in three-color channels. Three-color imaging was critical in our study to (1) distinguish tracers from lipofuscin granules, which are characteristic of aged brains^84^ (Figure S1F), and (2) to delineate AAV-infected cell bodies around the injection site, which was only possible in the unsaturated blue channel (ch^3^ Figure S1D). In our setup, the pixel spacing for x and y were 1.385 μm/pixel and 1.339 μm/pixel, respectively. Both hemispheres were sectioned concurrently in the coronal plane and in the rostral-to-caudal direction. We imaged four optical planes (12 μm intervals) per 50 μm slice for the rostral part and one optical plane per slice for the caudal part. We used only the first optical plane in this study. Complete sectioning of each brain was carried out in 20 – 30 sessions with different XY coverage, and it took approximately 10 days to process the whole brain.

#### Post-STPT histology

Tissue sections were manually collected for further histological analysis. To confirm the cytoarchitecture, we used one in ten sections for Nissl staining. The floating sections were mounted onto a glass slide after the agarose was removed and air-dried. Mounted sections were later rehydrated with PBS, and dark-field backlit images were taken using an all-in-one microscope (Keyence BZ-X710) before Nissl staining. The backlit image showed tissue contrast very similar to a conventional histological myelin stain (manuscript in preparation). We used the same section for Nissl staining, and the combined information of the backlit and Nissl-images provided useful information for anatomical delineation (Figure S3). AAV2retro-Cre was detected by anti-cre recombinase (Milipore, clone 2D8), followed by Cy3-conjugated secondary antibody. Retrograde nuclear staining was imaged by the all-in-one microscope and the images obtained were processed by the image processing pipeline for retrograde signal detection.^26^

For confocal microscopy, we stained the retrieved sections with the anti-GFP antibody (abcam, ab13790) and anti-Homer1 antibody (Nittobo Medical; previously FRONTIER INSTITUTE, MSFR103200) in conjunction with Alexa488 conjugated anti-chick antibody and Cy3-conjugated anti-rabbit secondary antibody. After mounting on a glass slide, the stained sections were imaged using an Olympus Fluoview FV3000 confocal microscope and a 40× silicone immersion lens (UPLSAPO40XS).

#### Image processing pipeline

The details of the image processing pipeline are described elsewhere.^26,85^ Briefly, the raw images in the three channels were stitched after background correction. The Ch1 (red) image provided the tissue background image with some tracer bleed-through, and the Ch2 (green) image provided the tracer fluorescence and tissue background. In our setup, we observed very weak signals for Ch^3^ (blue), which helped identify AAV-transduced cell bodies that were not identifiable with other channels due to signal saturation. Using this information, the pipeline automatically determined the precise spread of AAV transduction for each injection. This spread was calculated to be 2.5 ± 1.3 mm^3^ (mean ± SD) in the STPT template space (see below). We also calculated the depth bias of each injection (Deep Layer Index) by dividing the number of transduced cell-positive voxels in the deep layers (layer level 1–20) by those in all layers of the flatmap stack (see below), as shown in Table S1. The correlation between injection volume and patch number, patch size and solitary ratio was 0.001, 0.447, and 0.466, respectively, and the coefficient between the deep layer index and each of these variables was 0.13, 0.05 and 0.33. The correlation between the deep layer index and the DL ratio of patches was 0.5 (Figure S10D); the differences across subregions in the DL ratio persisted after correction (Figure S10E).

The pipeline also accurately separated axon-specific fluorescence from the background based on the Ch1 and Ch2 images. On visual inspection, we encountered virtually no false positives (e.g., lipofuscin granules misidentified as axons) for this process. Axonal detection was quite sensitive, although it was usually partial, meaning that not all visually recognizable axon fibers were labeled as the positive signals. The stitched coronal section slices (more than 600) were placed in the 3D space based on the recorded stage co-ordinates and non-linearly registered in 3D to the standard reference space or STPT template (see below). In this data transformation, a unit isocube (‘STPT-voxel’) corresponds to a 50 μm × 50 μm square of one slice image (50 μm interval), whose signal intensity was determined by the sum of positive pixels (out of ~1400), each of which was weighted for fluorescent intensity by its 16-bit encoding. In this way, we secured a wide dynamic range of quantification and a high signal-to-noise ratio. All quantitation of anterograde labeling was based on the fluorescence-weighted axon signal values.

Before normalization (see below), the total sum of thus-defined signal intensities was highly variable between samples due to various experimental factors, including the variable spread of the tracer near the injection site. However, there was little correlation with age (r = −0.062; p = 0.69) or sex (p = 0.38, by a t-test), which we did not analyze further in the current study.

To evaluate the registration accuracy, we compared the registered Ch1 images of individual samples with the STPT template. More precisely, we determined the boundary positions separating the cortex, putamen, globus pallidus, and internal capsule for each sample along a line ROI on horizontally sliced images (Figure S1A). These boundaries were determined automatically by selecting the peaks of optical density changes near each target boundary (red arrows (i)-(v)). As shown in the whisker plot, the deviations were within a few STPT voxel units (corresponding to 50-μm isocubes). Although this evaluation does not guarantee accurate registration for all the voxel points of the entire brain, it demonstrates highly accurate registration for regions near high-contrast landmarks, such as the white matter.

#### Standard reference space (STPT template)

To facilitate 3D-3D registration between optically-based and MRI-based volumes, we generated a standard reference image for a marmoset brain based on iteratively averaged Ch1 images.^26^ We call this 50 μm-isocubic 3D image as the “STPT template.” To allow multimodal data integration, the initial averaging step was performed using BMA2019 Ex Vivo (Space 2), which is a 100 μm isocubic reference image based on a population average of 25 *ex vivo* MRI (T2w) contrasts.^86^ Nissl-based cortical annotation originally performed on a single brain^24,87^ was transferred to the STPT template and used without further adjustment.

#### Handling of 3D data for visualization and substructure analyses

After completing the pipeline, the tracer intensity data and AAV-transduced area data were registered to the STPT template by ANTs using a multi-scale affine image registration followed by a multi-scale SyN registration.^26,73^ As a similarity metric, we used normalized mutual information. These 3D data were converted to a NIFTI format (https://nifti.nimh.nih.gov/), and structures of interest could be easily excised from each registered image volume using the labelmap. The labelmap for the definition of substructures was manually annotated based on the image contrast of the STPT template (Figure S1B) using 3D Slicer^72^ [https://www.slicer.org/]. After excising the structures of interest, the tracer intensities were standardized to the maximum value within the substructure, converted to 8-bit or 16-bit, and saved as a tiff stack file. FluoRender^71^ was used to visualize 3D data for the presentation of images and movies. Striatal data sometimes included strong contaminating signals of passing axon fibers within the internal capsule and white matter. In such cases, we manually masked these regions using a 3D Slicer (https://www.slicer.org/) and performed the normalization again.

#### Conversion of cortex data into a flatmap stack and its normalization

The outer (pial) and inner (white matter) segmentation surfaces of the cerebral cortex were defined based on the image intensity of the STPT template and manually corrected when necessary (Figure S1B). A 167,082-vertex surface mesh from the STPT template with vertices approximately midway through the cortical sheet provided an initial geometric substrate for generating pial, white, and midthickness surfaces that were in topological correspondence with one another. Radial trajectories determined by a dense orientation field created by a heat propagation model were used to generate the pial surface via vertex migration to the outer cortical boundary, the white surface via vertex migration to the inner cortical boundary, and the midthickness surface via migration to the midpoint of the radial trajectory linking the pial and white vertices. Fifty equally spaced vertices were identified along each radial trajectory connecting the inner and outer surface vertices (Figure S1C, right hemisphere). The Brain/Minds cortical midthickness flatmap^86^ based on the same 167,082-vertex mesh as the above 3D surface was used to define the x-y coordinates of a flatmap representation; the z dimension was represented by the spacing between vertices along each radial trajectory in the 3D volume. ANTs nonlinear registration^73^ was then used to generate a deformation field that aligned the 50-layer array of vertices in the 3D cortical model to the corresponding set of vertices in the flatmap stack. The deformed cortical volume was then resampled using trilinear interpolation to generate a flatmap stack volume based on a 500 × 500 × 50 array of ‘flatmap-stack-voxels’ (fm-vox; Figure S1C, left hemisphere).^26,85,86^

To evaluate the relationship between tangential distances in the 3D model vs the flatmap stack, we placed seed points at the midthickness layer in the flatmap stack with 50 fm-vox spacings. These seed points were mapped to the STPT template space using the deformation field. Spheres of 15-STPT-vox radius were placed at each seed point in the STPT template space and then mapped back to the flatmap stack using the deformation field (Figure S1E). The mean cross-sectional area was 247 fm-vox^2^. Given that the cross-sectional area for the 15-STPT-vox sphere can be approximated by a circle with 15-STPT-vox radius (706.5 STPT-vox^2^), and one STPT-vox corresponds to 50-μm in the STPT template space, one fm-vox in the flatmap stack based on these values is on average 84 μm on a side.

To standardize the intensities of the cortico-cortical projection patterns of the flatmap stack, we prioritized the constancy of signals in the upper layers (levels 26–50) because the lower layers contained more diffuse and widespread signals, some of which may be passing fibers. We first averaged layer levels 26–50 of the flatmap stack to make a 2D flatmap in which columnar convergence could be detected as peaks of fluorescence intensity. Herein, one pixel corresponds to tangential components of one fm-voxel. We masked the injection area based on the MIP of all layers of the flatmap stack for the injection site segmentation, because fluorescence intensity is usually saturated and unreliable in this region. We then selected pixels with the top 0.1% of the intensity values out of those that constituted the 2D flatmap and set the minimum value of the selected pixels at 10,000. This standardization method provided very similar patterns across different intensity ranges within the same frontal group irrespective of the original tracer intensity. The same coefficient was used to standardize the flatmap stack. For the six-color overlay of different PFC groups in Figure 1I, the MIP images for layer level 26–50 from the same PFC subregions were MIP-merged to generate a 2D flatmap. The flatmaps of different groups were then overlaid with different colors in a “winner-take-all” style, in which the color of the strongest signals was displayed to avoid color mixing. For the six-color overlay in Figure S1H, the color was merged using “Merge Channels Function” of ImageJ, by which regions of overlap had added values with cap at 255.

To convert the tracer intensity data into a log-scale representation, the standardized values were log-transformed after adding 0.001 and thresholding by 0. For visualization, the log-transformed data were scaled to an 8-bit representation. We routinely used the Videen color palette (obtained from Connectome Workbench color palette; https://github.com/Washington-University/workbench) for pseudocolor intensity scaling.

#### Calculation of Wiring distance

To estimate the distances between an anterograde tracer injection site and projection targets in the cortex, we used a minimal cost path algorithm. Our approach involved finding the cheapest paths within the tracer signal image that connect the center of the injection site with each tracer-positive location in the cortex. To achieve this, we assigned a cost to each voxel in the marmoset brain. The cost of a path was calculated as the sum of all voxels it needed to traverse to connect the injection site with the target location. We determined the cost of a voxel based on whether it had tracer signal, its location in white or gray matter, and whether it was located in a location where fibers did not pass (i.e., outside the brain or in brain fluid). Voxels inside the tracer had the lowest cost, followed by those in white matter, while the costs for voxels located outside gray and white matter were set to infinity. We used the skimage route_through_array function from the scikit-image image processing toolbox^74^ to compute the minimum cost paths. All calculations were done in the STPT reference space, and the resulting connection distance field was mapped to a cortical flatmap stack.

To create the binary mask GWM, we combined all gray matter and white matter voxels. We used the serial two photon template image STPT, normalized to the interval [0,1], as the likelihood map for gray matter since it was bright in cell-dense areas. We also had a binary mask CM comprising cortical and subcortical regions, which we used to determine projection targets. Additionally, we used the cell density image CELL indicating the injection site, as well as the inversed normalized tracer image TR (1 for no signal, 0 for strongest signal), both scaled to the interval [0,1]. All images were scaled down to a 200 μm isotropic resolution.

To determine the injection site, we first calculated the center of gravity of the cell density map. We then identified all projection targets as voxels that overlapped with the CM binary masks, had no signal in the cell density map CELL (i.e., not in the injection site), and had a signal in the tracer image (part of the tracer signal).

We computed the cost for voxels with tracer signal as tr_in = (TR+TC*10) (voxels where TR<1), and the cost for the other voxels as tr_out = (TC*10 + 50) (voxels, where TR=1). In both cases, voxels located in gray matter were more expensive than those in white matter (term TC*10). For the latter case, we added a constant of 50 to increase costs. We found these values heuristically. To favor paths in voxels with bright tracer signals over voxels with low intensities, we added the inverted signal TR to the first term. We then created the total cost image W = GWM*(tr_in + tr_out), where we set all costs outside GWM to infinity.

We computed the cheapest paths for all pairs between the center of the injection site and the position of the target voxels, and then created a path distance image by calculating the path lengths and storing the value at the target voxel locations. Finally, we mapped the path distance image to a flatmap stack (Figure S2C).

#### Exponential Distance Rule

Having established the injection-to-target distances, we investigated the relationship between connectivity strength and wiring distance. In previous retrograde tracer studies, connectivity strength was determined by “fraction of extrinsic labeled neurons (FLNe),” that is, the number of retrogradely labeled neurons in a given area relative to the total number of labeled neurons. FLNe thus represents the normalized connection weight between the target areas and the source area.^31,32,88^ Alternatively, distance-connection relationships were determined by calculating the probability of projection distance (p(d)), based on the histogram of the number of the labeled neurons in bins of projection distance.^32^ The latter value is independent of area specification, but both values matched well in the marmoset study.^32^ In our calculation, we used the flatmap-based approach to investigate the distance-intensity relationship. For each injection sample, the distance map and the strength map were made; the distance map shows the distance of each tracer-positive fm-vox from the injection site (determined in 3D as described above) and the strength map shows the log_10_ value for the normalized tracer signal intensity. For normalization, the tracer intensity values for all layers were averaged and adjusted using the same standardization factor used for the upper layer flatmap (see above). Using these two maps, we were able to determine the relationship between connection strength and wiring distance at the voxel level. An important question was whether it follows the Exponential Distance Rule (EDR), as reported for the macaque and marmoset retrograde studies.^31,32,88^ EDR postulates p(d)=ce^−λd^, where p(d) is the probability of connection and d is the projection distance.^31^ At the-synaptic targets, the axon fibers tend to branch heavily (Figure S8F) leading to higher tracer signals per unit voxel. Thus, the tracer signal intensity in our dataset is considered to reflect the connection probability. As shown in Figure S2G, the histogram of distance calculated from summed p(d) approximately follows an exponential decay with decay rate λ = 0.27. This histogram and λ are surprisingly similar to those reported for marmoset retrograde data (λ = 0.3; see Figure 5C of Theodoni et al.).^32^ Since the macaque study calculated the decay rate λ based on FLNe, we also performed a similar area-based analysis. FLP or “fraction of labeled projection” is based on the sum of tracer signals of a given area relative to the total tracer signals and corresponds to the FLNe of the retrograde data. The scatter plot showing the log_10_FLP values of each area plotted against wiring distance (Figure S2J) shows considerable variability but has a slope λ = 0.27. The large variability is comparable to that reported for marmoset^32^ and macaque.^31^ Our data suggest that a part of this variability comes from subdivision differences (Figure S2K). This observation is not surprising, considering the projections of PFC areas towards remote association areas skipping primary somatomotor areas (e.g., dlPFCv). When we tested EDR with columnar patches, which represent strong signals (Figure S2M), our results were consistent with previous EDR estimates up to a distance of 10–12mm. At longer ranges, we found a substantially shallower slope (Figures S2L–S2O).

#### Detection and characterization of columnar patches in corticocortical projections

Columnar patches were detected as local maxima in the 2D flatmap generated by averaging the upper layers (layer levels 26–50) of the 3D flatmap stack, with the injection sites masked and the signal values standardized as described above. Averaging across all flatmap layers gave similar results but with about 10% fewer patches, because diffusely spread signals in the deep layers sometimes obscured patches in the upper layers. Local maxima in 2D were identified using the “find peaks” function of MATLAB 2019a, inspired by the Fast 2D peak finder^89^ (https://www.mathworks.com/matlabcentral/fileexchange/37388-fast-2d-peak-finder), MATLAB Central File Exchange. Briefly, the 2D image was Gaussian smoothed, and peaks larger than the defined minimum height values were searched in the x- and y-directions, pixel by pixel. Only 2D spots that peaked in both the x-and y-direction searches were identified as local maxima. If the peaks were detected in consecutive fm-voxels, we counted them as one. Thus, the theoretical minimum separation is ~1.41 fm-vox (2e0.5), which occurs when the detected peak voxels are in a diagonal position. The minimum height value for columnar patch detection was set at 100, i.e., two orders of magnitude lower than the standardization value of 10,000. Columnar patches detected at the fringe of the injection site due to masking were removed manually by visual inspection. We detected 1867 columnar patches from 44 datasets. In this algorithm, we set the filter size of the Gaussian smoothing to 7-fm-vox. Setting it to 3-fm-vox resulted in detection of only 10 % more patches, and most patches were identical. The additional patches were generally weak and surrounded by high background signals. We conclude that the current filter size effectively captures a patchy signal distribution while reducing noisy signals.

To examine the extent of convergence of tracer signals to the centers of columnar patches, we set three 3D ROIs for measurement (Figure 2C); namely, a central cylinder with a 4-fm-vox radius, a 19x19x50 fm-vox rectangular cuboid (hereafter referred to as 19-vox cuboid) and a 50x50x50 fm-vox cubic (hereafter referred to 50-vox cubic). The idea is to measure the ratio of the number of tracer-positive voxels contained within the cylinder to that in the 19-vox cuboid and the 50-vox cube. First, we expressed the intensity of the tracer signals in each voxel as a fraction of the maximum value within the upper half of the central cylinder. Second, the tracer signals were binarized at 0.5, half the maximum value. Third, the largest clump of the binarized tracer signals within the upper half of the central cylinder was chosen to represent the columnar patch of choice (Figures 2B and 2C, green “clump”). The ratio of the number of voxels within the central cylinder to that of all voxels that constitute this binary clump within the 19-vox cuboid and 50-vox cube was calculated as the index of central convergence. Columnar patches were often clustered, and stronger tracer signals were at nearby locations connected to the central clump. Therefore, we classified the columnar patches into “solitary” and “connected” groups based on the degree of central convergence. When we set 50% of central convergence for the 50-vox cubic ROI as the threshold, 1008 patches out of 1867 patches fulfilled this criterion. The central convergence for this group of patches was equal to that of the 19-vox cuboid ROI, which means that the segmented central clumps of the tracer signals were contained within the 19-vox cuboid and disconnected from the surrounding signals. Hence, we called this group “solitary”. The patches that did not fulfill this criterion were called “connected” because the central clumps for over 97 % of the remaining 859 patches extended outside the 19-vox cuboid. Here, the size of the central cylinder was adjusted to maximize the inclusion of columnar signals and minimize the inclusion of surrounding signals based on the planar average (Figure 2D). We reasoned that the presence of 50% of the voxels of the continuous binary clump in the 50-vox cubic ROI is a reasonably high convergence. The 19-vox cuboid ROI was used as a middle ROI to confirm the connectedness of the clump. Because of the relatively high degree of central convergence, the morphology of the central clumps for the solitary groups could be reasonably well approximated by ellipsoids whose major axis lengths, centroid positions, and angles were estimated by regionprop3 function (CY Y; 2022;. https://github.com/joe-of-all-trades/regionprops3).

To estimate the size of the patches, we measured the tangential spread of the above-mentioned binary clumps by projecting the positive voxels across all layers. We measured only the solitary patches to exclude inclusion of surrounding signals. We also measured the ratio of the solitary patches among all the patches for each injection. We expected this value to indirectly reflect the patch size by becoming smaller when the patches get bigger and connect to the surrounding signals. Indeed, we found a tendency of dmPFC, FP and ACC patches to show larger solitary patch sizes and lower solitary ratios (Figures S4A–S4C). In rare cases, detected patches that were in close proximity were aggregated when measuring the patch size. We did not adjust for such cases.

#### Comparison of mouse and marmoset data for columnar projections

All the mouse data used in this study were obtained from Mouse Brain Connectivity Atlas^28,29^ (https://connectivity.brain-map.org/). First, we selected the datasets with injections into the frontal areas (ACAd, ACAv, FRP, ILA, MOs, ORBI, ORBm, ORBvl, PL) of the wild-type mice. The experimental IDs for these samples are; 112458114, 139426984, 146593590, 112514202, 139520203, 100140756, 157556400, 100141454, 112952510, 141602484, 141603190, 157710335, 180709942, 180719293, 180916954, 585025284, 112306316, 170721670, 180673746, 180709230, 126860974, 112423392, 158435116, 157711748. The whole-brain tracer data (projection energy) with 10 μm resolution for these datasets were then acquired via the application programming interface (API) and converted to flatmaps as previously described.^90^ The flatmaps for both hemispheres were resized to 816×408 pixels. The image intensity was standardized similarly to that used for the marmoset flatmap except that the injection center was not excised before measurement. The columnar patches were detected using the same code used for the marmoset flatmap. The mouse flatmaps represented the average of all layers, whereas the marmoset analysis had used flatmaps representing only the upper half of the layers. For comparison, we performed a new patch detection using the flatmaps of all layers. As shown in Figure S6, the columnar patches were far less conspicuous for inter-areal mouse PFC projections; the strong within-area patchiness observed for marmosets (e.g., robust columnar projections near the injection site in Figures S6E and S6F) occurred rarely in mice. This difference was quantitatively shown (Figure S6G).

Our detection algorithm simply finds the peaks above a threshold in the X and Y directions after smoothing. It can therefore detect broad peaks that do not necessarily extend radially like those of the marmoset cortex. It is also sensitive to standardization methods. The mouse data differ from our marmoset data in several ways. (1) The mouse study used regular AAV-GFP, which is less efficient at labeling axons than our TET-enhanced AAV. (2) Mouse brains were sliced at 100 μm intervals as opposed to 50 μm intervals for our marmoset brains, and is accordingly less reliable in detecting submillimeter scale structures. (3) The frontal region of the mouse is curved, and flatmapping causes large distortions. (4) We could not subtract the tracer signals of the cell bodies before standardization for the mouse data. All these differences might in principle result in differences in the detectability of columnar patches in the two species. However, the original slice images suggest that the projection patterns of axons are very different between mice and marmosets (e.g., Figures S6B and S6F). Therefore, we consider the large species differences in the incidence of patches (Figure S6G) to be a robust finding.

#### Prediction of columnar patch distribution patterns by polynomial regression for corticocortical projections

To examine the topographic projections to the cingulate and temporal fields, we set the ROIs for these regions as shown in Figure 2G, and determined the center of mass of the columnar patches present within each ROI for each sample for the six PFC subregions. Among the 29 injections, 24 had at least one columnar patch in the cingulate field, and 21 had columnar patches in the temporal field. To find a regression model to predict the projection from the injection, we used a polynomial regression of degree 2, which exhibited a better corrected AIC score than degree 3. As predictor variables, we used the X and Y coordinates of the injection center in the flatmap. As response variables, we used the X or Y coordinates of the averaged positions of columnar patches in the cingulate and temporal ROIs. As shown in Figures S4D and S4E, we achieved reasonable fitting (R^2^ =0.68~0.92) for the injections in PFC. The permutation test, in which the models constructed by the original and shuffled data were compared for accurate reconstitutions, confirmed the effectiveness of the regression model. In this test, the accuracy of the predictions by the regression models was estimated by correlation coefficients between the predicted values and the true values. Comparison of the model constructed by the original dataset with those by 1000 shuffled datasets showed that none of the shuffled models surpassed the accuracy of the original data (p<0.001). To extrapolate the obtained topography, we calculated the projections from every point in the PFC map to the cingulate and temporal fields, as shown in Figure 2H. This calculation resulted in projections outside the flatmap, suggesting the presence of distortions at the fringes. Figure 2H displays only the projections within the flatmap region.

#### Analysis of the frontal patch distribution pattern

We set the extent of frontal cortex to be the boundary between motor and somatosensory cortex posteriorly and between OFC and insular cortex laterally (see Figures 2G and 2I). To quantify the distribution of the columnar patches in the frontal areas, we used ellipse fitting on the flatmap representation. Because the columnar patches tended to show a distorted distribution along the narrowly stretched area 24a, we excluded this area from ellipse fitting (see Figure S5B for ROI). As shown in Figure S5B, the centers of the ellipses were generally offset from the injection centers, but the relative positions were maintained. This relationship was approximated by polynomial regression models of degree 2 (Figure S5C). The permutation test similar to what we did for the patch center prediction (see the preceding section) confirmed the robustness of the regression models (p<0.001). To visualize the spread of columnar patches generated from injection into different PFC subdivisions, ellipses that encompassed 70 % of the patches for each injection were overlaid and the common overlapping region for over half (>49%) of the injection samples was used as an indicator of the spread of frontal projections for that subdivision (enclosed by thick colored lines in Figure S5B).

To analyze the spacing of patches, we examined the shortest inter-patch distance for each patch that was within the ellipse for that injection and calculated the mean for each injection sample (Figures S5D–S5G). These measurements were based on distances within the flatmap and can differ modestly from the 3D distance due to distortion, particularly for ACC patches, as medial and orbital areas that are widely separated in the flatmap are substantially closer in the 3D configuration. Overall, we observed generally similar values for the mean of the shortest interpatch distance across samples (~1mm). This value was statistically indistinguishable from the values for randomly shuffled controls (x1000 repetitions, data not shown). In a previous study, a center-to-center distance of 500–600μm was reported as the spacing of intrinsic connectivity in macaque PFC.^13^ Our value is slightly larger but still in reasonable agreement, considering the very different labeling and detection methods and the species difference.

#### Analysis of contralateral projection patterns

To evaluate the bilateral symmetry of projection patterns, we compared the mirror-flipped images of the contralateral projections with the ipsilateral images. For 2D comparison, we used the mean values for the upper layers (26–50). As expected from previous reports of “homotopic” contralateral projections,^91,92^ we observed very similar patterns of the log_10_ images for both hemispheres after intensity adjustment (the right intensity was 12.8 ± 7.8 % of the left intensity). To quantitatively evaluate the similarity of strong projections around the injection site, we set a 200×200 fm-vox ROI surrounding the injection site for each sample for the left (ipsi) and right (contra) flatmaps. We excluded the cell-positive injection regions from both ipsi and contra images and binarized the images to extract the top 5 % of the tracer-positive voxels. This made the patterns of tracer distribution comparable between contra and ipsi sides and across samples. To evaluate the similarity between ipsilateral and contralateral patterns, we compared the overlap of the true pairs with shuffled pairs and found that the true pairs showed greater overlap than shuffled pairs in all but three cases, in which some shuffled pairs showed higher overlap than the true pair (Figure S5K).

#### Non-negative matrix factorization (NMF) analyses of corticocortical and corticostriatal projection patterns

To find common patterns of projections for corticocortical (or corticostriatal) projection, we performed NMF analyses using log-transformed data. NMF is an effective method of dimensionality reduction for neuroimaging data.^93^ Unlike principal component analysis, all coefficients and basis images are nonnegative, allowing a more straightforward interpretation of the results.^94^ For corticocortical projections, 500 × 500 fm-vox MIPs of all layers with injection site masks were downsized to 100 × 100 size, and the data for 44 injections were converted to a 44 × 10,000 matrix to be used for “nnmf” function of MATLAB. For corticostriatal projections, a 180 × 300 × 220 STPT-vox space containing the left striatum was downsized to a 45 × 75 × 55 space, and a 44 × 185,625 matrix was used for NMF analyses. NMF analysis attempts to decompose the original matrix A (sample × pattern) into basis images (or components) W and coefficients H (A ≅ WH). Given the nonnegative constraints, A is not exactly equal to WH. Furthermore, the nnmf function of MATLAB uses an iterative algorithm starting with random initial values for W and H, and the results obtained vary each time slightly. To find the best result, we repeated the analyses 100 times and examined the residuals between A and WH. The residuals were relatively constant but sometimes became large. Inspection of the basis images showed that very similar images were generated when the residuals were small. Therefore, we selected W and H with the smallest residuals as the basis images. Because we had only 44 datasets for analysis, we tried to minimize the number of basis images while achieving adequate reconstitution efficiency. With four basis images, the averaged correlation coefficients between the reconstituted and the original images were approximately 0.927 (Figure S8B). Reconstitution by five basis images did not result in substantial improvement (0.934), and we chose to use four basis images for further analysis.

Conversion of NMF_W1, 2, 3, and 4 into a single color map was performed by first combining the W1/W4 and W3/W2 pairs by subtraction. By this subtraction, W1 (or W3)-dominant regions take positive values, whereas the W4 (or W2)-dominant regions take negative values because of the nonnegative feature. Furthermore, because of the antagonistic relationship of these pairs, overlapping regions that cancel each other’s value are small in extent. As a result, the positional information of the four components (or basis images) was mostly retained in the positive and negative domains of the subtracted values (compare Figure 3B with Figure 3D). Using the 2D colormap shown in Figure 3G,^75^ these two value sets can be jointly represented by color-coding. This color-coded map contains only information about the ratio of the two coefficient differences and not about coefficient magnitude. In the associated spatial map (Figure 3H), regions with a low contribution of any of the four components were excluded. Conversion of coefficients 1, 2, 3, and 4 into a colored dot map (Figure 3H) was performed similarly.

#### Analysis of retrograde labeling

We used AAV2retro-EF1-cre as a non-fluorescent retrograde tracer. This construct accumulates CRE protein in the nucleus, which results in relatively even labeling of neurons of diverse sizes. It was also advantageous for the automatic identification of labeled cells. After immunohistological detection and imaging, the retrieved image data were processed for automated segmentation of labeled nuclei and registration to the corresponding STPT image (FR and HS, manuscript in preparation). Although we collected only one in ten sections to detect the retrograde tracer, we were able to map the result of retrograde tracing to the STPT template by registering the slice images to the STPT slice data. To evaluate the colocalization of anterograde and retrograde tracers at the columnar scale, we measured the image intensity by “Plot Profile” function of FIJI using the vertical line ROI shown in Figure 5B^70^ [https://imagej.net/software/fiji/]. We also measured the amount of each tracer in the 117 cortical areas of the flatmap stack format for the examination of correlations. The correlation coefficients in Figures 5G and S11 were calculated based on the log_10_ of the original signal values. We did not adjust the signal values for the size of each area and we excluded areas with fewer than threshold (10^0.5^=~3.2) retrograde signals from calculations of correlation coefficients.

Visual inspection of antibody-stained images revealed some injection cases with a low signal-to-noise ratio, as judged by an abundance of false-positive artifacts across multiple cortical areas. We distinguished such samples by calculating the ratio between the number of areas having low values and the number with above-threshold values, excluding areas with no signals. The samples that failed this quality check exhibited lower correlation coefficients (Figure S11B), suggesting that a high signal-to-noise ratio of the retrograde tracing data, which is determined by the sensitivity and specificity of immunolabeling, affects the correlation with the anterograde data. In two cases, we also observed substantial leak of the retrograde AAV into the white matter, which appeared to have transduced the cells via passing fibers. We excluded these two cases from further analyses.

Although the anterograde and retrograde tracer distributions were well correlated overall, there were mismatches of several types. Some mismatches were attributable to purely technical confounds or differences in methodology. Technical confounds included gaps in data arising from the wider interval between sections analyzed for retrograde data and also misregistration, especially in distorted regions on the fringes. In addition to these technical reasons, methodological differences could lead to mismatches. One significant difference is that each retrogradely labeled cell is counted as one, irrespective of the amount of tracer contained in the nucleus. We suspect that the diffuse connectivity is less efficiently labeled by the retrograde tracer and that its identification is more sensitive to experimental conditions. On the other hand, our anterograde tracing cannot distinguish passing fibers from synaptic connections and may overestimate genuine “connectivity”, especially for sparse signals. Thus, the evaluation of reciprocity using the dual tracer system that we adopted in this study requires careful consideration of each case.

#### Lamina-profiling of the columnar and diffuse cortical projections

Inspection of signal distributions for individual columnar patches indicated various laminar preferences for these patches. Because the orientation of axonal profiles was not always orthogonal to the flatmap but was largely contained within the central cylinder of 4-fm-vox radius, we measured the maximum intensity of tracer signals within the central cylinder for each of the 50 layers. We defined this as the laminar profile of the columnar patch of interest. We performed hierarchical clustering to classify the laminar profiles of the 1867 columnar patches. Pearson’s correlation coefficient was used to measure distance, and Ward’s method was used for the clustering. Although we selected columnar patches based on the values of layer level 26–50, the profiles of all 50 layers were used to calculate the correlation coefficient. Figure 4A shows the original laminar profiles of the columnar patches (before averaging) aligned according to the tree structure of the hierarchical cluster analysis. We divided them into eight clusters (separated by white border lines) and averaged each of the laminar profiles shown in Figure 4A. We further defined three lamina types, “DL,” “ML”, and “UL”. The defined lamina types were color-coded blue, green, and red, respectively, for representation in flatmap format in Figure S10B.

To examine the laminar profiles of the overall areal projections, including both the columnar and diffuse projections, we divided the cortical hemisphere into 117 areas and examined the averaged laminar profile in each area. When we calculated the sum of tracer signals for the 44x117 injection-to-target area pairs, 97% of the pairs had non-zero signals. To assess the significance of these signals, we aligned the log_10_ values of the sum of the signals in order and found that the decline in log_10_ values gradually accelerated around the 4,000^th^ pair and dropped sharply after the ~4,500^th^ pair. Accordingly, we decided to use the top 4,000 injection-to-target area pairs for laminar profiling as the pairs with significant connections. The hierarchical clustering was performed in a similar manner to that used for the focal projections, except that we made a new group called “DL2”, which showed very restricted signal distribution near the gray/white matter interface. Some of these signals may represent fibers of passage. At present, we cannot distinguish between synaptic contacts and fibers of passage, although we did observe bouton-like varicosities to be associated with some deep-layer axons by confocal microscopy (Figure S8F).

#### Occupancy and colocalization of focal (patchy) tracer signals in the striatal region

We noticed that the occupancy of tracer signals in the striatal regions varied widely among the PFC subregions when focal/patchy projections were visualized (Figure 6A, linear view). To quantitatively estimate this feature, we set the threshold at 50% of the maximum intensity (Figure 6C) and counted the ratio of positive voxels in the caudate nucleus, putamen, and nucleus accumbens, separately over the entire striatal voxels for each sample. The values in the three striatal compartments were summed for analysis by ANOVA to compare the six PFC subregions. For the colocalization analysis, the ratio of the overlapping voxel numbers to the number of combined voxels was calculated for every pair of 29 injections (Figure S12C). Due to the patchy distribution, overlap within the same PFC subregions was low on average but varied greatly across pairs.

#### Detection of patches of corticostriatal projections in 3D

For detecting patches of corticostriatal projections in 3D, the 3D data were first converted to a set of XY and YZ MIP images for the detection of local maxima in each 2D image in a similar way used for the detection of corticocortical columnar patches, and the 3D positions that fit both MIP images were selected as the local maxima in 3D. When two patches were detected within a six-STPT vox distance in 3D, we merged them for simplicity. The detected patches were projected onto the XY, YZ, and XZ MIP images for visual inspection and accurate detection. In the detection of caudate patches, we found that strong signals in the Muratov bundle or internal capsule were sometimes included and selected as patches. In such cases, we either masked those regions for re-standardization or simply deselected them. Tracer intensities were standardized to the maximum values within the striatal region for each sample, and the minimum height value for patch detection was set at half the maximum value. An example of such patch detection is shown in Figure S12D. The distribution of these patches could generally be well approximated by an elongated ellipsoid (Figure S12D, red ellipses), consistent with the previous observation that macaque corticostriatal projections are longitudinally aligned.^41^

#### Prediction of corticostriatal patch distribution patterns by polynomial regression model

To find regression models that can predict the projection coordinates from the injection coordinates, we tested the polynomial regression model with degree 2 and searched for the optimal fit. As predictor variables, we used the x, y, and z coordinates of injections in the STPT template space. As response variables, we used the x, y, or z coordinates for the average positions of the detected STPT-vox intervals that roughly correspond to AP = +13.5, +14.5, +15.5, +16.5, and +17.5 in the Paxinos atlas and visualized the corresponding lines in the caudate.

### QUANTIFICATION AND STATISTICAL ANALYSIS

#### Statistical tests

We generally used 8, 6, 5, 2, 4, and 4 samples for dlPFCv, dlPFCd, dmPFC, FP. ACC, and OFC, respectively, for subregion comparisons. The injections that were positioned near the borders of these subregions were excluded in such cases. Values are reported as mean ± standard deviation (SD) throughout the manuscript. The correlation coefficients (r) in Figures 5G, 5H, S8B, and S11 refer to the Pearson correlation. Those in Figure 6H refer to Spearman’s rank correlation. R^2^ for the regression model refers to the coefficients of determination in Figures S4D, S4E, and S8C. The p-values were calculated using one-way ANOVA and Tukey’s post hoc test for significant factors in the ANOVA for Figures 2E, 3C, 6D, and 6G.

## Supplementary Material

4

2

1

3

## Figures and Tables

**Table:** KEY RESOURCES TABLE

REAGENT or RESOURCE	SOURCE	IDENTIFIER
Antibodies		
anti-cre recombinase (clone 2D8)	Milipore	RRID:AB_2085748
anti-GFP antibody (ab13790) Abcam		RRID:AB_2936447
anti-Homer1 antibody (MSFR103200)	Frontier Institute	RRID:AB_2571774
Bacterial and virus strains		
AAV1 -Thy1S-tTA	Mizukami Laboratory	N/A
AAV1 -TRE-clover	Mizukami Laboratory	N/A
AAV1 -TRE3-Vamp2-mTFP1	Mizukami Laboratory	N/A
AAV2retro EF1-Cre	Mizukami Laboratory	N/A
Biological samples		
Marmoset brain tissue	This paper	N/A
Deposited data		
Marmoset tracer data This paper		https://dataportal.brainminds.jp/marmoset-tracer-injection.
Mouse Brain Connectivity Atlas	Harris et al.^28^ and Oh et al.^29^	https://connectivity.brain-map.org/
Recombinant DNA		
pAAV-Thy1StTA	Sadakane et al.^68,69^	RRID:Addgene_97411
pAAV-TRE3_Clover	This paper	RRID:Addgene_135179
pAAV-TRE3_ mTFP1- Vamp2	This paper	Addgene plasmid # 201214 (processing)
pAAV-EF1_Cre	This paper	Addgene plasmid # 201198 (processing)
Software and algorithms		
MATLAB (2019a)	MathWorks	https://www.mathworks.com/products/matlab.html
ImageJ/Fiji	Schindelin et al.^70^	https://imagej.net/software/fiji/
FluoRender	Wan et al.^71^	https://www.sci.utah.edu/software/fluorender.html
3D Slicer	Fedorov et al.^72^	https://www.slicer.org/
ANTs	Avants et al.^73^	http://stnava.github.io/ANTs/
scikit-image	Van der Walt et al.^74^	https://scikit-image.org/
Other		
2D colormap	https://github.com/dominikjaeckle/Color2D; Steiger et al.^75^	ziegler

## Highlights

- A whole-brain map of marmoset PFC projections has been created for open access
- Two projection types (patchy and diffuse) were identified in cortex and striatum
- Tightly reciprocal connections with column-scale precision were discovered
- Interregional connectivity is topographically arranged both globally and locally
