# Supplementary material for: Local and long-distance organization of prefrontal cortex circuits in the marmoset brain
Source: Neuron. Author manuscript; Available in PMC 2024 Jan 16. (PMC10789578; doi:10.1016/j.neuron.2023.04.028)
Supplement: 4 [file NIHMS1956534-supplement-4.pdf]

Neuron, Volume *111*

Supplemental information

Local and long-distance organization of prefrontal cortex circuits in the marmoset brain

Akiya Watakabe, Henrik Skibbe, Ken Nakae, Hiroshi Abe, Noritaka Ichinohe, Muhammad Febrian Rachmadi, Jian Wang, Masafumi Takaji, Hiroaki Mizukami, Alexander Woodward, Rui Gong, Junichi Hata, David C. Van Essen, Hideyuki Okano, Shin Ishii, and Tetsuo Yamamori

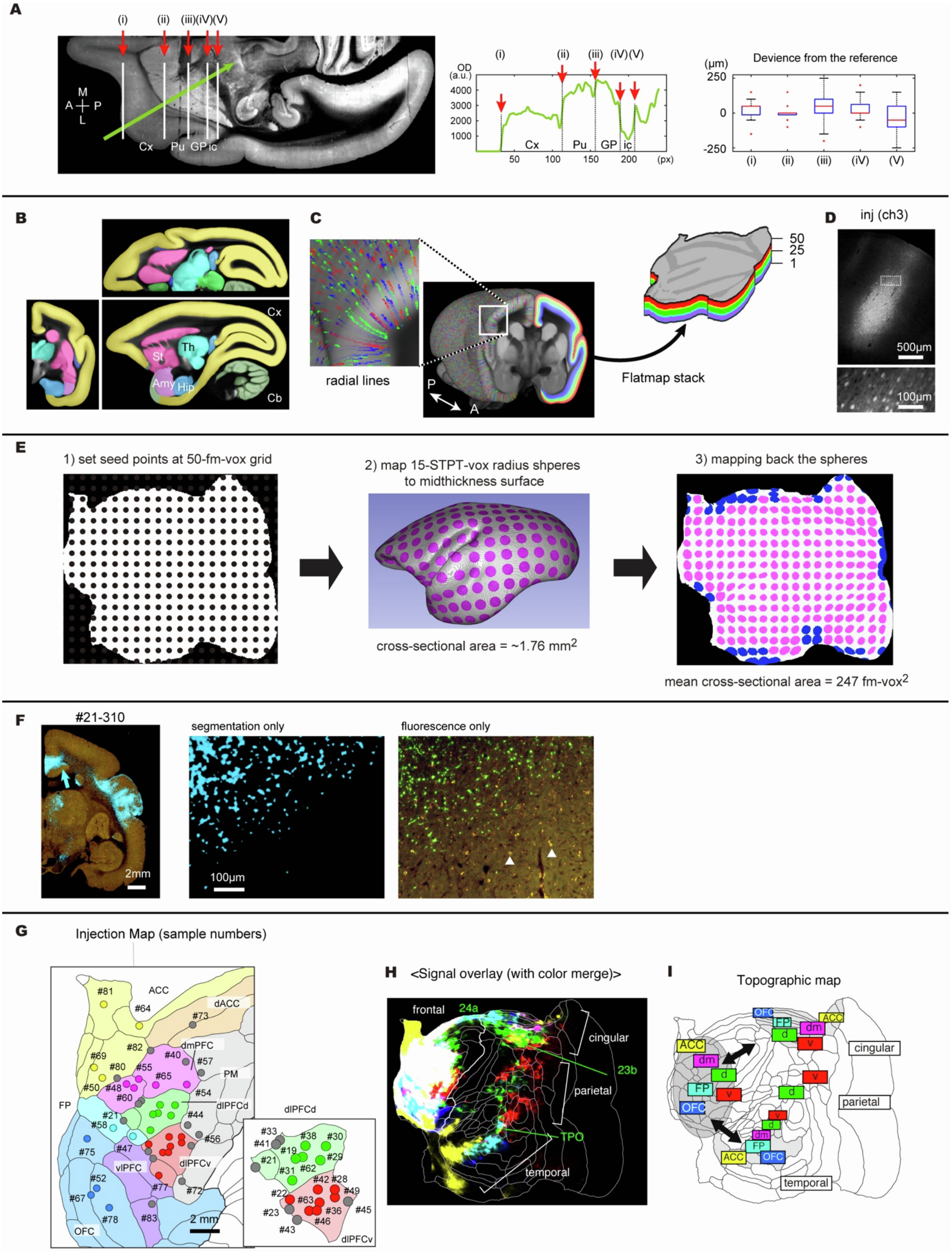

A
(i) (ii) (iii)(iV)(V)
M A P L
Cx Pu GP ic
OD (a.u.)
4000 3000 2000 1000
Cx Pu GP ic
50 100 150 200 (px)
(μm)
Devience from the reference
250 0 -250
(i) (ii) (iii) (iV) (V)
B
Cx St Th Amy Hip Cb
C
radial lines
P A
Flatmap stack
50 25 1
D
inj (ch3)
500μm
100μm
E
1) set seed points at 50-fm-vox grid
2) map 15-STPT-vox radius shperes to midthickness surface
cross-sectional area = ~1.76 mm2
3) mapping back the spheres
mean cross-sectional area = 247 fm-vox2
F
#21-310
2mm
segmentation only
100μm
fluorescence only
G
Injection Map (sample numbers)
ACC dACC dmPFC PM FP dlPFCd dlPFCv vlPFC OFC
2 mm
H
<Signal overlay (with color merge)>
frontal 24a cingular 23b parietal TPO temporal
I
Topographic map
OFC FP dm ACC d v cingular parietal temporal

Figure S1, Related to Figure 1: Mapping of PFC projections by serial two-photon tomography imaging (STPT). (A) An example image showing how we estimated registration accuracy along one dimension (green line). The 3D-reconstructed images were registered to the STPT template and virtually sliced in the horizontal plane. Only the right hemisphere is shown (A; anterior, P; posterior, M; medial, L; lateral). The image intensity (optical density; OD) of individual samples was measured along the line ROI, which revealed the anatomical borders (i)-(v) separating the cortex (Cx), putamen (Pu), globus pallidus (GP), and internal capsule (ic). The deviation from each border determined for the STPT template is shown by box and whisker plots on the right. Note that 50 μm corresponds to one pixel. px; pixel. **(B)** Coronal, parasagittal, and horizontal section views for the STPT template overlaid with anatomical annotations. Cx, cortex; St, striatum; Th, thalamus; Amy, amygdala; Hip, hippocampus; Cb, cerebellum. **(C)** Schematic sparse representation of the radial lines that determined the columnar structures of the cortical surface in different colors (right hemisphere) and the lamina structures determined by these vertices (left hemisphere). This information was used to convert the cortical surface into a stack of 50 layer-specific flatmaps. **(D)** The bleed-through fluorescence in the blue channel reveals the exact site of the injection [inj(ch3)]. Note that the fluorescence of individual cells can be recognized, unlike in saturated channels 1 (red) and 2 (green) (see Figure 1A and 1C). **(E)** A schematic explanation for how we measured the tangential intracortical distance in the flatmap stack. We first placed a grid of seed points on the midthickness layer in the flatmap stack at interval of 50 flatmap-stack-voxels (fm-vox). These seed points were mapped to the 3D STPT template space and spheres with 15-STPT-voxel radius were centered at each seed point. These spheres were mapped back to the flatmap stack using the deformation field. Finally, cross-sections of the deformed 15-voxel sphere in the mid-layer flatmap were used to calculate the approximate correspondence of 15-voxel distance in the flatmap stack. Any spheres that intersected the outer boundary of the flatmap or to an adjacent sphere were excluded from calculation (shown by blue color). **(F)** Comparison of tracer segmentation (cyan) and original fluorescence. Aged marmoset brain sections typically contain many lipofuscin granules with dot-like autofluorescence [S1] (white arrowheads in the right panel), which was efficiently excluded in our signal detection algorithm. **(G)** Estimated locations of injection sites in relation to six PFC subregions: dlPFCd (dorsolateral PFC-dorsal), green; dlPFCv (dorsolateral PFC-ventral), red; dmPFC (dorsomedial PFC), magenta; FP (frontopolar cortex), cyan; ACC (anterior cingulate cortex), yellow; OFC (orbitofrontal cortex), blue. Others include vlPFC (ventrolateral PFC), dACC (dorsal anterior cingulate cortex), and PM (premotor areas), in which injection points are shown by gray dots. These colored injections were used as core samples for various analyses. Depending on the analyses, gray injections located outside the six PFC subregions (e.g., premotor areas) or on the borders between subregions were also used. See Supplementary Table 1. The uncertainty in localization in the tangential domain (parallel to the cortical sheet) is difficult to determine, but is likely to be within one mm, considering the projection patterns of each sample. The scale bar shows the approximate distance on the midpoint flatmap based on panel (E). **(H)** The overlay of projection patterns for six PFC subregions that allows color mixing. The convergence of projections from different subregions in A24a, A23b, and TPO, as well as most frontal areas, resulted in color mixing. Same as in Figure 1I, except for color mixing. **(I)** A schematic representation of the topographic projections deduced from the overlay of tracer signals for the six PFC subregions shown in Fig. 1I. d; dlPFCd, v;dlPFCv, dm; dmPFC.

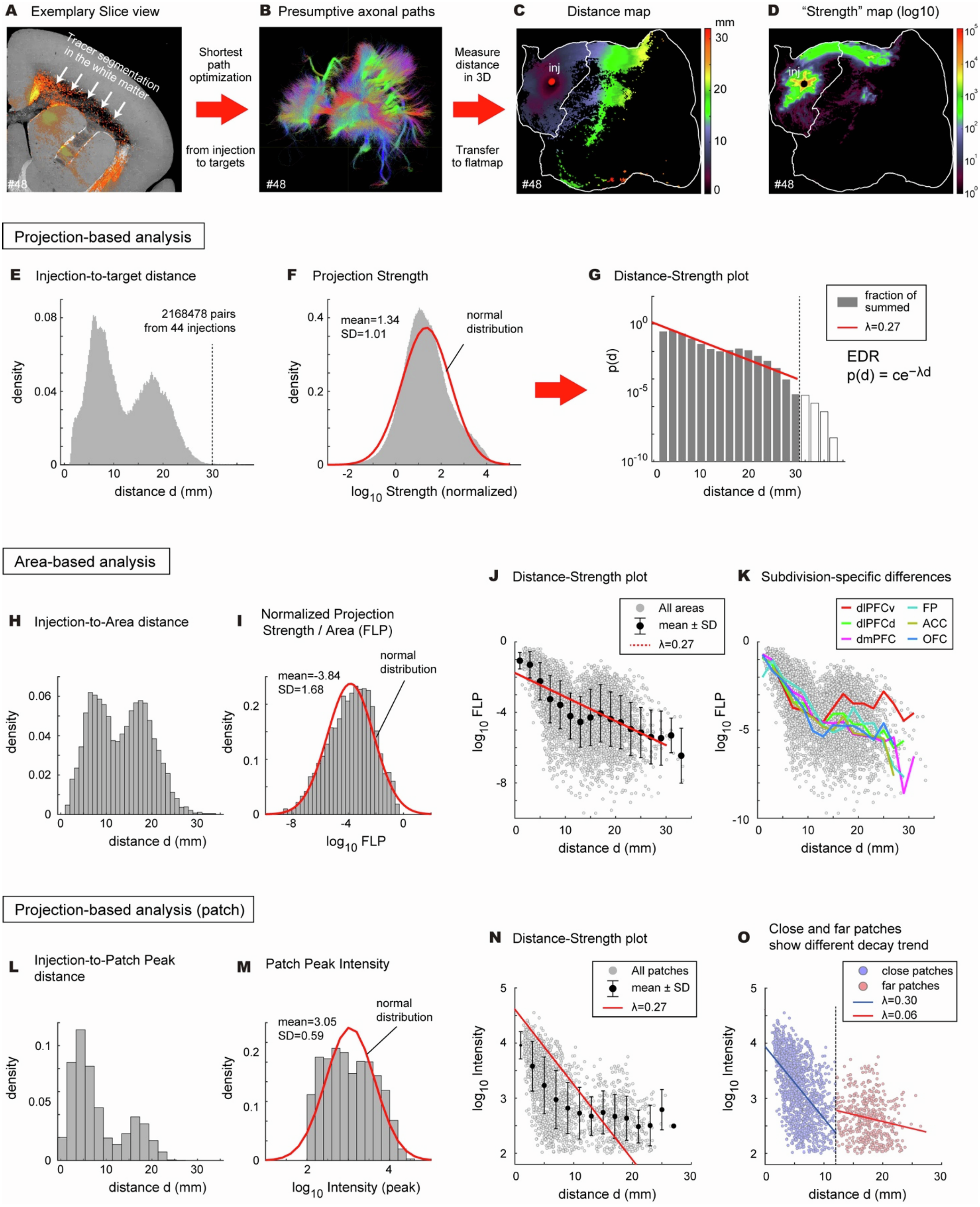
A Exemplary Slice view
Tracer segmentation in the white matter
#48
Shortest path optimization
from injection to targets
B Presumptive axonal paths
#48
Measure distance in 3D
Transfer to flatmap
C Distance map
inj
#48
mm
D "Strength" map (log10)
inj
#48
Projection-based analysis
E Injection-to-target distance
2168478 pairs from 44 injections
density
distance d (mm)
F Projection Strength
mean=1.34
SD=1.01
normal distribution
density
log10 Strength (normalized)
G Distance-Strength plot
fraction of summed
λ=0.27
EDR
p(d) = ce−λd
p(d)
distance d (mm)
Area-based analysis
H Injection-to-Area distance
density
distance d (mm)
I Normalized Projection Strength / Area (FLP)
mean=-3.84
SD=1.68
normal distribution
density
log10 FLP
J Distance-Strength plot
All areas
mean ± SD
λ=0.27
log10 FLP
distance d (mm)
K Subdivision-specific differences
dlPFCv
dlPFCd
dmPFC
FP
ACC
OFC
log10 FLP
distance d (mm)
Projection-based analysis (patch)
L Injection-to-Patch Peak distance
density
distance d (mm)
M Patch Peak Intensity
mean=3.05
SD=0.59
normal distribution
density
log10 Intensity (peak)
N Distance-Strength plot
All patches
mean ± SD
λ=0.27
log10 Intensity
distance d (mm)
O Close and far patches show different decay trend
close patches
far patches
λ=0.30
λ=0.06
log10 Intensity
distance d (mm)

Figure S2, Related to Figure 1: Distance-projection strength relationships for diffuse and patchy projections. (A-C) Strategy to measure the distance between the injection and the projection sites. **(A)** An exemplary slice view to show the tracer segmentation within the white matter. The signals in the grey matter are partially blocked to highlight the white matter signals. **(B)** Shortest path optimization of the axon paths from the injection site to various cortical projection targets based on white matter signals (see STAR Methods for detail). **(C)** Distance map, a flatmap representation of the distance from the injection site to various locations in the flatmap. Note that these values represent distance measured in the STPT template 3D space. **(D)** "Strength" map, which shows the $\log_{10}$ of the normalized tracer intensity (see STAR Methods) in color scaling as a surrogate for connection strength. **(E-G)** Projection-based analysis determined the relationship between the distance and connection strength for each location in the flatmap. **(E)** Distribution of the injection-to-target distance for 2168478 nonzero pairs from all the 44 samples. This is not lognormal and includes two prominent peaks. **(F)** Distribution of the projection strength from all the samples. The red plot indicates the normal distribution expected from the same mean and SD. They are similar, but not identical. **(G)** The fractions of summed projection strength plotted against distance. The bin size is 2 mm. The red line is a linear fit to the $\log_{10}$ values of the summed strength for the distance between 0 and 30 mm. The values above 30 mm were not used because the sample size is too small [see panel (E)]. We used the sum of the projection strength to calculate the decay trend (λ) on the assumption that signal intensity reflects the number of axons, and thus, connection strength. Exponential Distance Rule (EDR) postulates $p(d)=ce^{-\lambda d}$, where p(d) is the probability of connection and d is the projection distance. In our data, we obtained $\lambda=0.27$, which is strikingly similar to the value obtained by the retrograde tracer data [S2]. **(H-K)** Area-based analysis to determine the relationship between the distance and connection strength on the injection-to-area basis. For this analysis, we calculated FLP (fraction of labeled projection) in a similar manner to FLNe for the retrograde data [S3], by calculating the sum of tracer intensities for a given area divided by the total tracer intensities for each injection sample. **(H and I),** Again, injection to target area distance showed two peaks and the $\log_{10}$ FLP value showed slightly distorted normal distribution pattern. **(J)** $\log_{10}$ FLP as a function of interareal wiring distance. Black dots and error bars are the mean and SD (bin size is 2 mm) and the red plot indicates the $\lambda=0.27$. Note the high variability, which is also observed in the marmoset and macaque retrograde data [S2, S4]. **(K)** The means for the different subdivisions were plotted on the same scatter plot as in panel (J), suggesting subregion differences in decay trend. **(L-O)** Projection-based analysis to determine the relationship between the distance and connection strength for the columnar patches. **(L)** Similar to the above analyses, we observed two peaks for the distance distribution. **(M)** The peak intensity exhibits only two orders of magnitude in its ranges because of thresholding for patch detection. **(N)** Distance-strength plot as in panel (J). The patches appear to consist of two different populations with different decay trends. **(O)** Linear fitting was used for close (<12 mm) and far (>12mm) patches. The decay trends for these two populations were very different ($\lambda=0.30$ and 0.06, respectively).

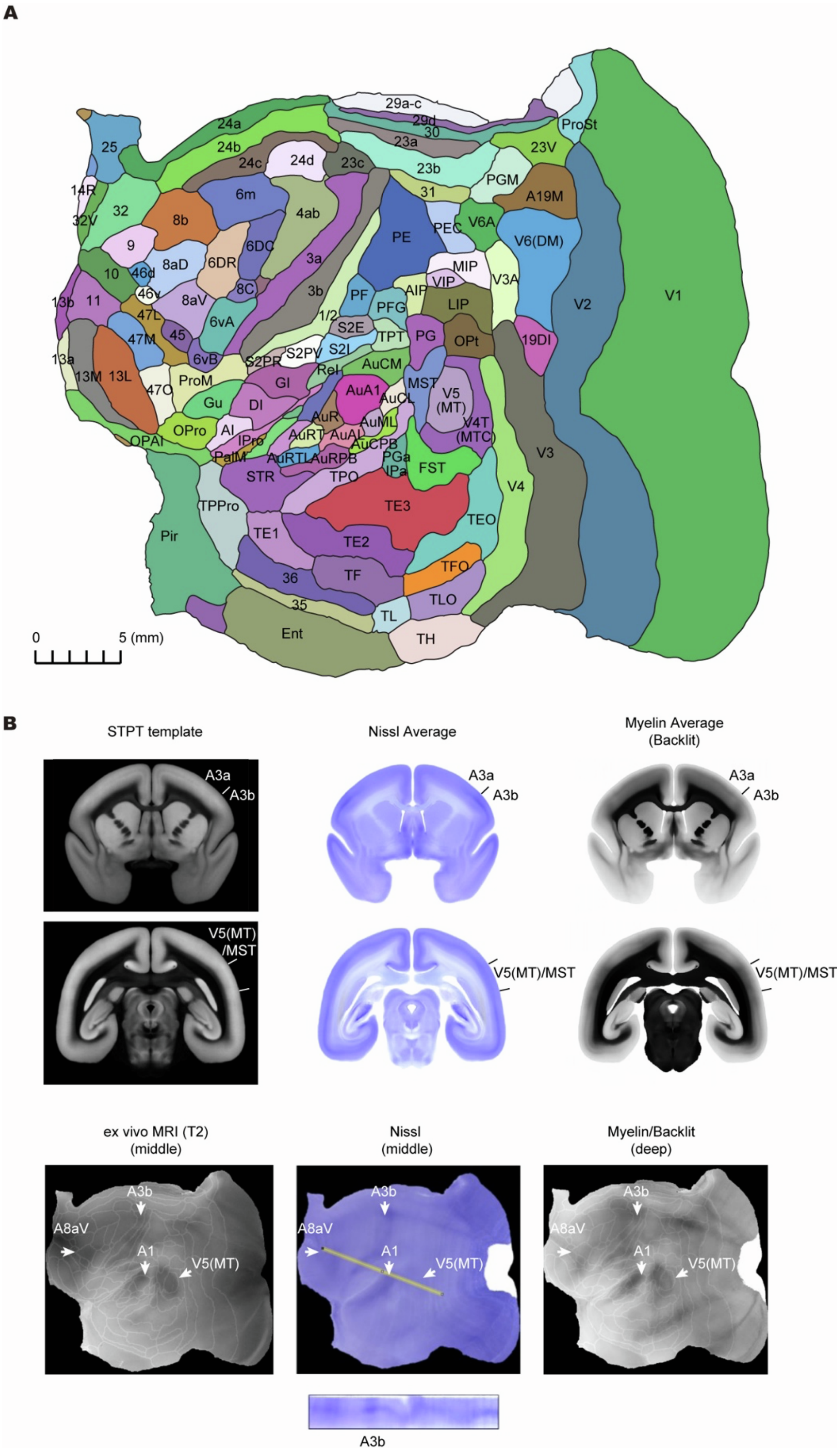

Figure S3, Related to Figure 1: A map of the cortical areas and comparison with histological data. (A) The cortical area annotation of the Brain/MINDS atlas [S5, S6] is transferred to the standard reference space (STPT template) and the flatmap stack for registration. The scale bar shows the approximate distance on the midpoint flatmap based on Figure S1E. **(B)** Comparison of the cortical annotation of the STPT template with histological data. After STPT imaging, one in ten sections was retrieved for Nissl staining and backlit myelin imaging (see STAR Methods). Here, the section and flatmap views for the averaged Nissl and backlit (myelin) images are shown. Several conspicuous landmarks in these images corresponded well to cortical annotations.

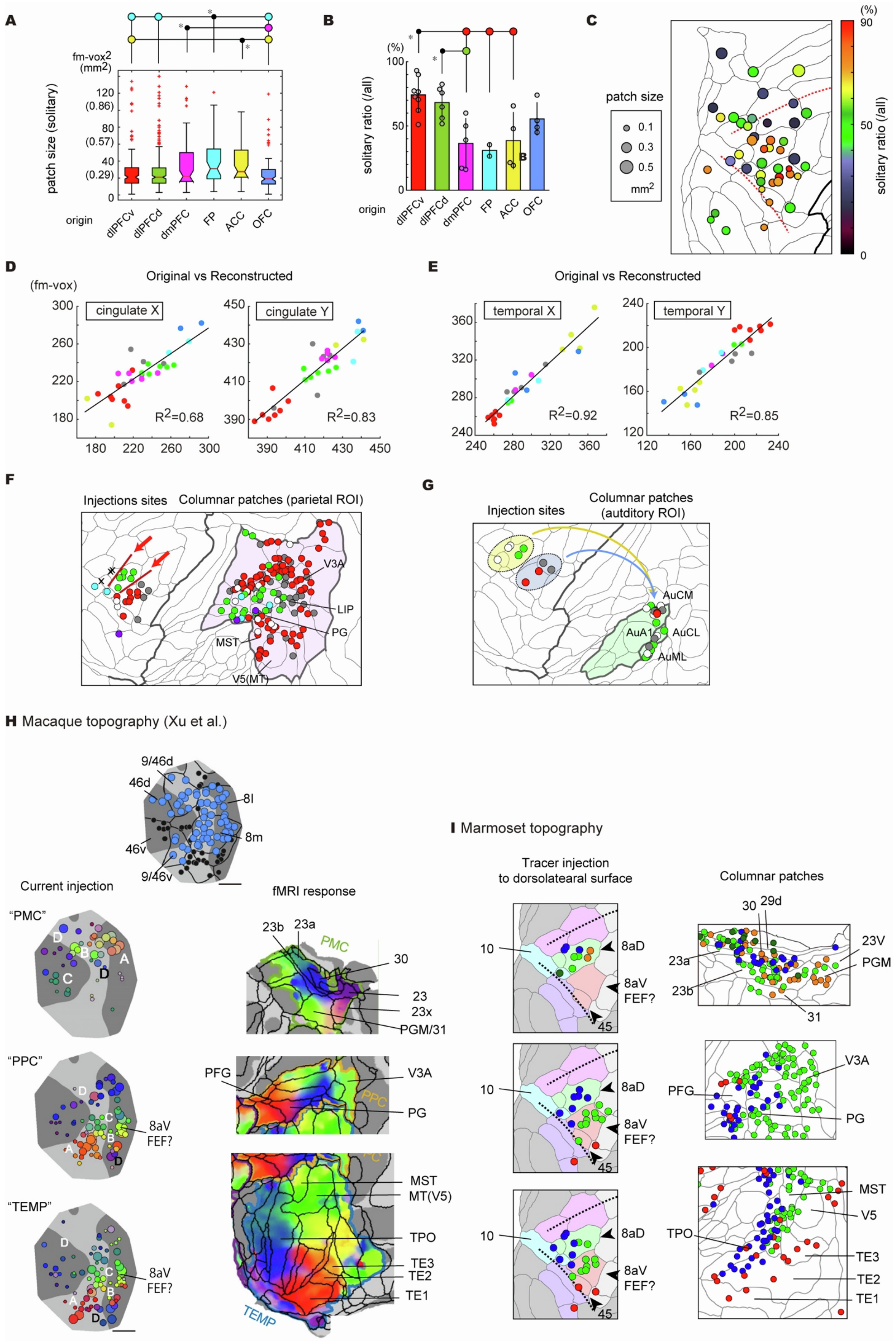
A
B
C
D
E
F
G
H Macaque topography (Xu et al.)
I Marmoset topography
patch size (solitary)
solitary ratio (/all)
origin
dlPFCv
dlPFCd
dmPFC
FP
ACC
OFC
patch size
0.1
0.3
0.5
mm2
Original vs Reconstructed
cingulate X
cingulate Y
temporal X
temporal Y
R2=0.68
R2=0.83
R2=0.92
R2=0.85
Injections sites
Columnar patches (parietal ROI)
V3A
LIP
PG
MST
V5(MT)
Injection sites
Columnar patches (autditory ROI)
AuCM
AuA1
AuCL
AuML
Current injection
"PMC"
"PPC"
"TEMP"
fMRI response
9/46d
46d
8l
8m
46v
9/46v
8aV
FEF?
23b
23a
PMC
30
23
23x
PGM/31
PFG
PG
PPC
MST
MT(V5)
TPO
TE3
TE2
TE1
TEMP
Tracer injection to dorsolatearal surface
Columnar patches
10
8aD
45
29d
23V
PGM
31
V5

Figure S4, Related to Figure 2: Topographic organization of the columnar patch distribution. (A) Comparison of the solitary patch size originating from six PFC subdivisions. The colored circles indicate statistically significant differences (ANOVA with Tukey's honest significant difference test, $p<0.05$) between the subregions of these colors and the subregions under these circles. The size is shown by fm-vox area and also by calculated mm^2 scale. The result suggests differences associated with regions of injections. We did not observe significant differences associated with the regions of projections except for slightly larger size for OFC (data not shown). **(B)** Comparison of solitary ratio (solitary/solitary+connected) for each injection in six PFC subdivisions. The statistical significance is as explained for panel (A). Bar; SD. **(C)** The mean size of the solitary patches (in circle diameter) and the ratio of the solitary patches /all patches (shown by color scale) for each injection are displayed. **(D) (E)** Comparison of original and reconstructed values from polynomial regression models for topographic projections. The colored dots represent the injections into six PFC subregions, and the gray dots represent the border injections. **(F)** Seed-based analyses to determine injections that exhibited columnar projections to the parietal field. The colors of the injection sites and the columnar patches in the target ROI indicate which subregion they belong to (FP, cyan; dlPFCd, green; dlPFCv, red; vlPFC; purple; PM, gray; white, border). The red arrows and lines indicate borders of abrupt changes in projection profiles. The injections indicated by X did not generate columnar patches in the parietal field, demonstrating an abrupt border. **(G)** Seed-based analyses to determine injections that exhibited columnar projections toward the early auditory field (core and belt regions). Projections from the anterior and posterior PFC regions intermingled in the auditory field, unlike the parietal field. **(H)** Connectivity mapping of the macaque lateral PFC by stimulation-fMRI, reproduced from Xu et al [S7] with permission. Stimulation of the colored dots in the lateral PFC generated responses in the target regions having matched colors. Nomenclature for the cortical areas is as reported in Xu et al. **(I)** Tracer injections in the marmoset PFC generate columnar projections with topographic relationships similar to the macaque counterparts. Injections into the colored dots generate columnar patches with the same colors in the target regions. Nomenclature for the cortical areas is as shown in Figure S3. Note that the colors used for this panel differ from other figures to highlight similarity with the macaque data.

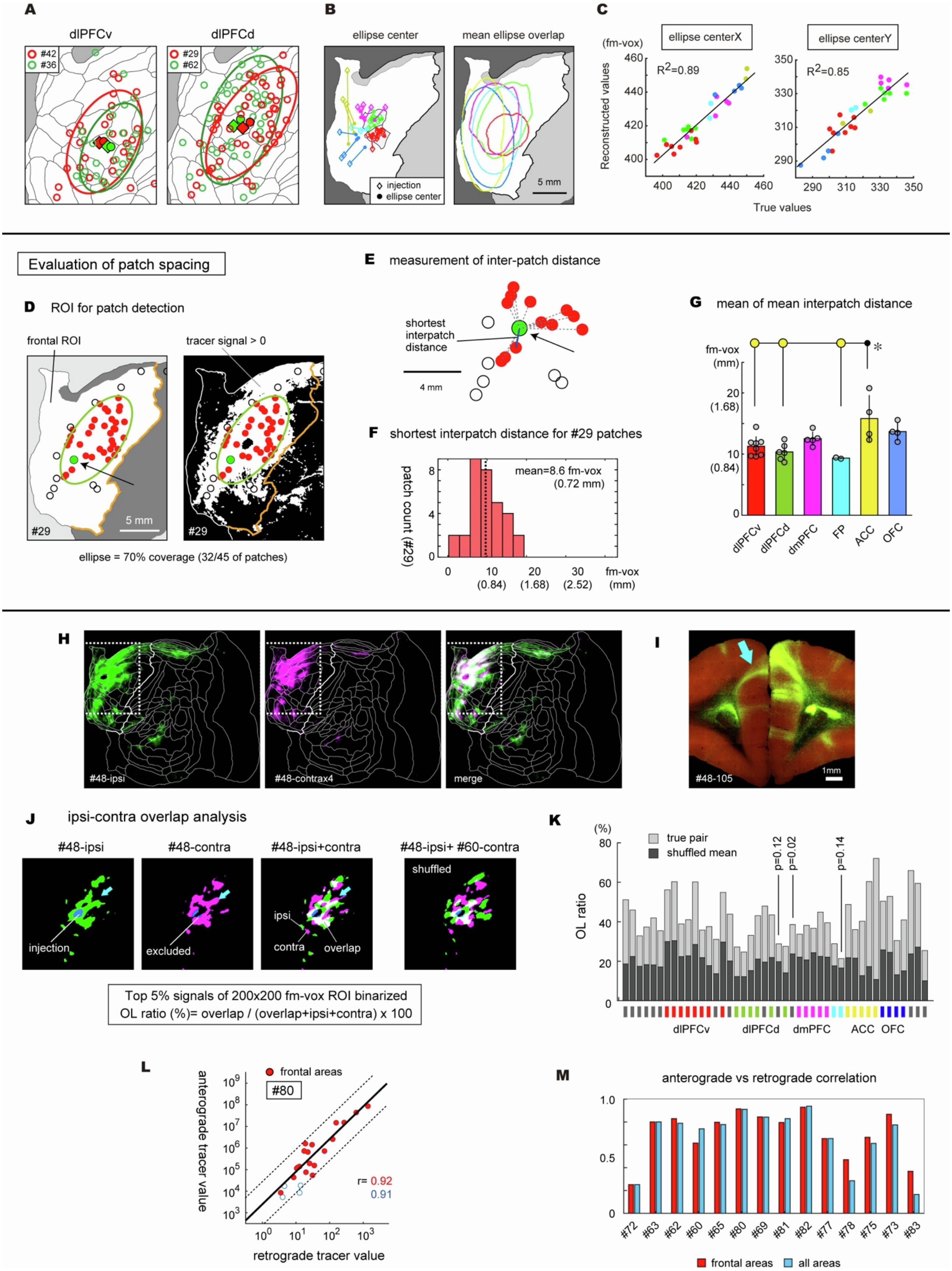
A
dlPFCv
dlPFCd
#42
#36
#29
#62
B
ellipse center
mean ellipse overlap
injection
ellipse center
5 mm
C
(fm-vox)
ellipse centerX
ellipse centerY
R^2=0.89
R^2=0.85
Reconstructed values
True values
Evaluation of patch spacing
D ROI for patch detection
frontal ROI
tracer signal > 0
5 mm
#29
ellipse = 70% coverage (32/45 of patches)
E measurement of inter-patch distance
shortest interpatch distance
4 mm
F shortest interpatch distance for #29 patches
mean=8.6 fm-vox (0.72 mm)
patch count (#29)
fm-vox (mm)
G mean of mean interpatch distance
fm-vox (mm)
dlPFCv
dlPFCd
dmPFC
FP
ACC
OFC
H
#48-ipsi
#48-contrax4
merge
I
#48-105
1mm
J ipsi-contra overlap analysis
#48-ipsi
#48-contra
#48-ipsi+contra
#48-ipsi+ #60-contra
injection
excluded
ipsi
contra
overlap
shuffled
Top 5% signals of 200x200 fm-vox ROI binarized
OL ratio (%)= overlap / (overlap+ipsi+contra) x 100
K
(%)
true pair
shuffled mean
p=0.12
p=0.02
p=0.14
OL ratio
dlPFCv
dlPFCd
dmPFC
ACC
OFC
L
frontal areas
#80
anterograde tracer value
retrograde tracer value
r= 0.92
0.91
M
anterograde vs retrograde correlation
#72
#63
#62
#60
#65
#80
#69
#81
#82
#77
#78
#75
#73
#83
frontal areas
all areas

Figure S5, Related to Figure 2: Columnar patch distributions in ipsilateral and contralateral cortex. (A-G): ipsilateral frontal cortex. (A) A pair of nearby injection sites in dlPFCv (left panel) and a different pair of nearby injection sites in dlPFCd (right panel). Note scattering of abundant columnar patches around the injection site, whose overall extent is approximated by ellipses enclosing 70 % of the patches. The injection sites (filled circles) and ellipse centers (diamonds) were slightly offset, but the topographic relationship between the two adjacent injections was preserved. The spread of patches was more restricted for this dlPFCv pair than the dlPFCd pair. (**B)** (Left panel) The offsets between the injection sites (diamonds) and the ellipse centers (filled circles) for each sample of the six core PFC subregions are shown. These patterns reveal a wide dispersion of columnar patches from peripherally-located ACC and OFC injection sites (yellow and blue traces). (Right panel) The regions enclosed by colored lines represent regions occupied by the ellipses of at least half of the samples (>49 %) of each subregion. The overlap of ellipses for each subregion was smallest for dlPFCv (red trace). **(C)** Polynomial regression to fit the positions of the ellipse centers based on the injection coordinates. **(D)** [Left panel] ROI for analysis of patch spacing. The patch distribution of the white area in the frontal field was analyzed. The ellipse represents the 70 % coverage of the patches present in this ROI. **(E)** The shortest interpatch distance was defined for each patch within the ellipse (e.g., green patch indicated by the arrow) by calculating all the combinations within the ellipse. **(F)** The histogram showing the shortest interpatch distance for the 32 patches of sample #29. The measurement is based on the flatmap stack (fm-vox based). The approximate correspondence of fm-vox values in mm scale is shown by parentheses. **(G)** The means of the mean interpatch distance among six PFC subdivisions are compared. We observed a modest but statistically significant difference between ACC and three other subregions. This result suggests approximately 1 mm spacing (for dmPFC) for patch separation, which is somewhat larger but compatible with intrinsic stripe spacing for macaque frontal projections (500-600 μm) [S8], considering difference of species and methods. **(H-M): Contralateral cortical projections. (H)** An example overlay of the ipsi- (green) and contra- (purple) lateral cortical projections. Contralateral projections are mirror-flipped. Contralateral projections are generally much weaker than the ipsilateral projections. To achieve similar visibility, the intensities were adjusted by four-fold. The dotted boxes indicate parts of 200x200 fm-vox ROI used for overlap analysis in panel (J). **(I)** A coronal section view indicating obvious asymmetry of the left-right patterns (cyan arrowhead). **(J)** Strategy to estimate the symmetry of ipsi/contra projections. 200x200 fm-vox ROIs were excised from the flatmaps for the ipsi- (left) and contralateral (right) cortices centered on the injection coordinates. The top 5% of the tracer signals within these ROIs were binarized to test overlaps. The injection sites were excluded from comparison. The blue-shaded areas in the ipsi and contra images represent excluded tracer signals. The Overlap ratio (OL ratio) was defined by the percentages of the overlapping areas divided by the combined areas of ipsi- or contralateral tracer signals. As a control, we examined the OL ratios of a given sample with contra-images of other samples (shuffled control). The cyan arrows indicate the position of apparent asymmetry shown in panel (I). **(K)** OL ratios for the same (true) ipsi/contra pairs compared with the mean of the shuffled controls (dark). Except for three samples as denoted, true pairs were always higher than the shuffled controls ($p<0.05$). **(L)** Area-based comparison of anterograde and retrograde signals for the contralateral projections as in Figure 5G. The correlation coefficients (r) for the frontal areas only and for all areas combined are shown in red and blue lettering, respectively. **(M)** Correlation coefficients of the retrograde and anterograde signals for the contralateral projections in the frontal areas only (red bars) and for all areas (blue bars). Note that the contralateral signals are generally weaker and sparser than the ipsilateral signals. This can result in a small dynamic range of intensity distribution in some cases, contributing to reduced correlation.

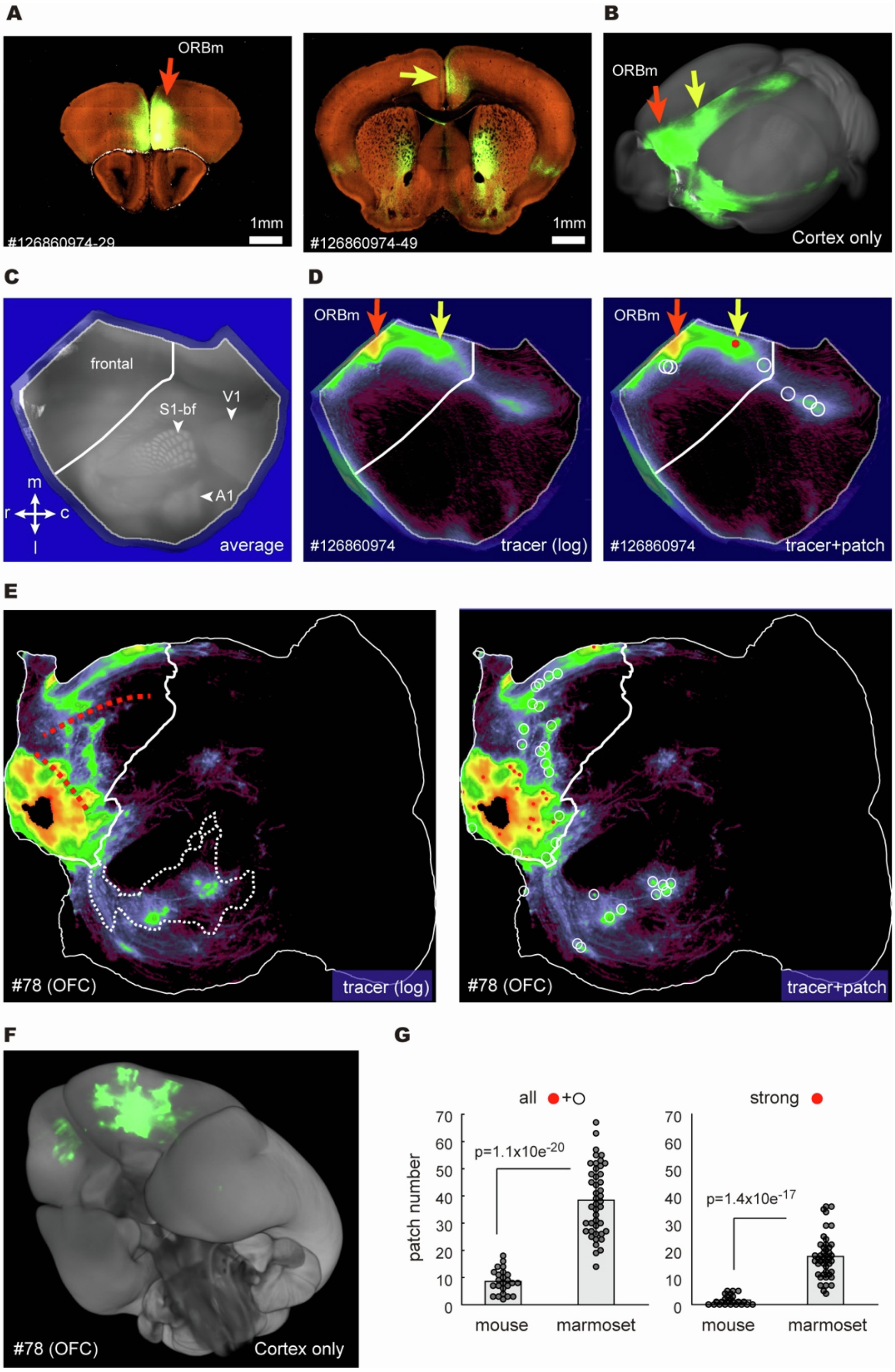
A
ORBm
1mm
#126860974-29
1mm
#126860974-49
B
ORBm
Cortex only
C
frontal
S1-bf
V1
A1
m
r
c
l
average
D
ORBm
#126860974
tracer (log)
ORBm
#126860974
tracer+patch
E
#78 (OFC)
tracer (log)
#78 (OFC)
tracer+patch
F
#78 (OFC)
Cortex only
G
all
strong
patch number
p=1.1x10e-20
p=1.4x10e-17
mouse
marmoset
mouse
marmoset
0
10
20
30
40
50
60
70

Figure S6, Related to Figure 2: Comparison of mouse and marmoset data for patchy projections.
(A) Left: A coronal section centered on an injection site (red arrow) in ORBm (126860974) retrieved from the Mouse Brain Connectivity Atlas. Right: coronal section through a local maximum detected as in panel (D) (shown by a yellow arrow). **(B)** Translucent 3D reconstructed view of the cortical projection data. **(C)** Flatmap image for the averaged background fluorescence for cortical landmarks. Owing to spatial distortions, the fringes of the flatmap were excluded from patch detection. The middle white line indicates the border between the motor and somatosensory areas. S1-bf; S1 barrel field. m; medial, l; lateral, r; rostral, c; caudal. **(D)** The flatmap image of the ORBm injection in logscale was pseudocolored to show both strong and weak signals. Red dots and white circles on the right indicate columnar patches detected with our algorithm with peak values that are >1/10 and >1/100 respectively of the standardization value (see STAR Methods for details). **(E)** Detection of columnar patches for marmoset injection #78 (OFC) using the mean of all layers for comparison with the mouse data. The white line in the middle indicates the border between motor and somatosensory areas. The red and white dotted lines in the left panel indicate the dorsomedial/ventrolateral convexities (ridges) of the cortex and the temporal areas, respectively (see Figure 2I). **(F)** Translucent 3D reconstructed image of the cortical projection data for case #78 viewed from the bottom. **(G)** Comparison of detected patch numbers per injection between the mouse and the marmoset. The right panel compares only the strong signals (shown as red dots). See STAR Methods for information on the mouse data (24 samples) used in this analysis. All 44 samples were used for the marmoset data.

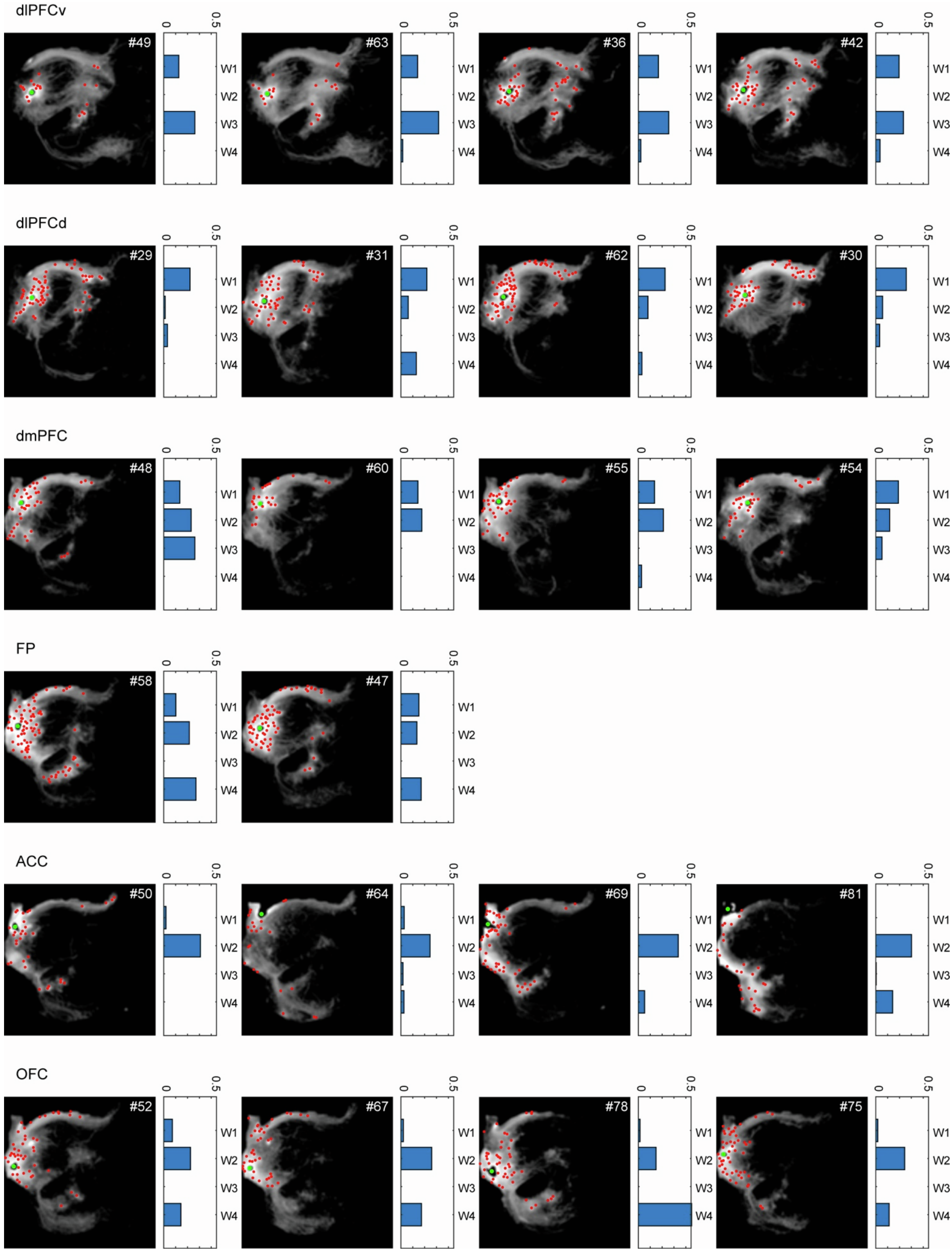

Figure S7, Related to Figure 3: The log-scale projection patterns for individual injections explained by NMF weights. Selected examples of log-scale tracer images, representing the diffuse projections organized into six PFC subdivisions. The coefficients for the four NMF basis images are shown right for each sample. Note similarity of the projection patterns and the coefficient patterns within the same subdivisions.

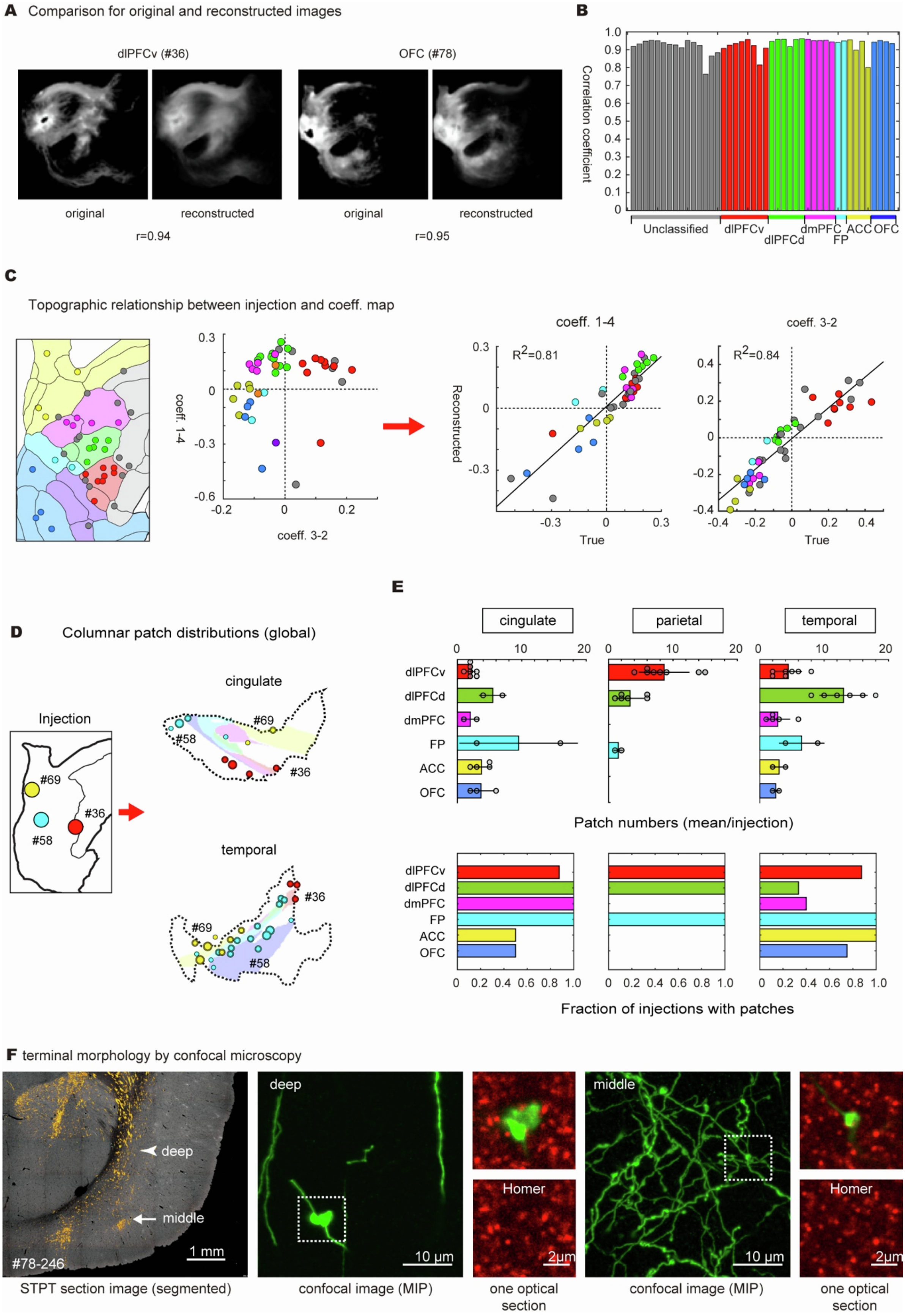
A Comparison for original and reconstructed images
dlPFCv (#36)
OFC (#78)
original
reconstructed
r=0.94
r=0.95
B
Correlation coefficient
Unclassified
dlPFCv
dlPFCd
dmPFC
FP
ACC
OFC
C
Topographic relationship between injection and coeff. map
coeff. 1-4
coeff. 3-2
Reconstructed
True
R2=0.81
R2=0.84
D
Columnar patch distributions (global)
Injection
cingulate
temporal
#69
#58
#36
E
cingulate
parietal
temporal
dlPFCv
dlPFCd
dmPFC
FP
ACC
OFC
Patch numbers (mean/injection)
Fraction of injections with patches
F terminal morphology by confocal microscopy
deep
middle
#78-246
1 mm
10 μm
2μm
Homer
STPT section image (segmented)
confocal image (MIP)
one optical section

Figure S8, Related to Figure 3: Characterization of diffuse corticocortical projections in log-scale view. (A) Comparison of the original images and those reconstructed from the four NMF components for two exemplar injections. **(B)** The accuracy of the reconstruction was measured using the correlation coefficients of the two images for 44 pairs. **(C)** Comparison of topographic relationships of injection sites with plot positions in the coeff. 1-4 vs coeff. 3-2 value map. The polynomial regression models (degree 3) could predict these coefficients based on injection coordinates as shown. **(D)** Global gradients affect the patch numbers and intensity for the columnar projections. Three example injections into dlPFCv (#36), FP (#58), and ACC (#69) generated columnar patches both in the cingulate and temporal association fields in a topographic manner, but the numbers and intensity greatly differed. The size of the circles represents the normalized intensity of each patch. **(E)** [upper panel] The numbers of patches in cingulate, parietal and temporal fields for each injection in different PFC subdivisions. Some injections did not generate columnar patches in these fields (e.g., dmPFC, ACC and OFC in parietal cortex). [lower panel] Fraction of injections having at least one patch in the indicated target region. Together, panel (E) suggests the presence of global gradients for the formation of columnar patches. **(F)** Morphological examination of tracer signals by confocal microscope. Tracer signals are examined in the deep (arrowhead) and middle (arrow) layers. The corresponding sections were retrieved for staining with anti-GFP and anti-Homer antibodies (a postsynaptic marker). The deep layer (arrowhead) and middle layer (arrow) regions imaged by a confocal microscope are shown.

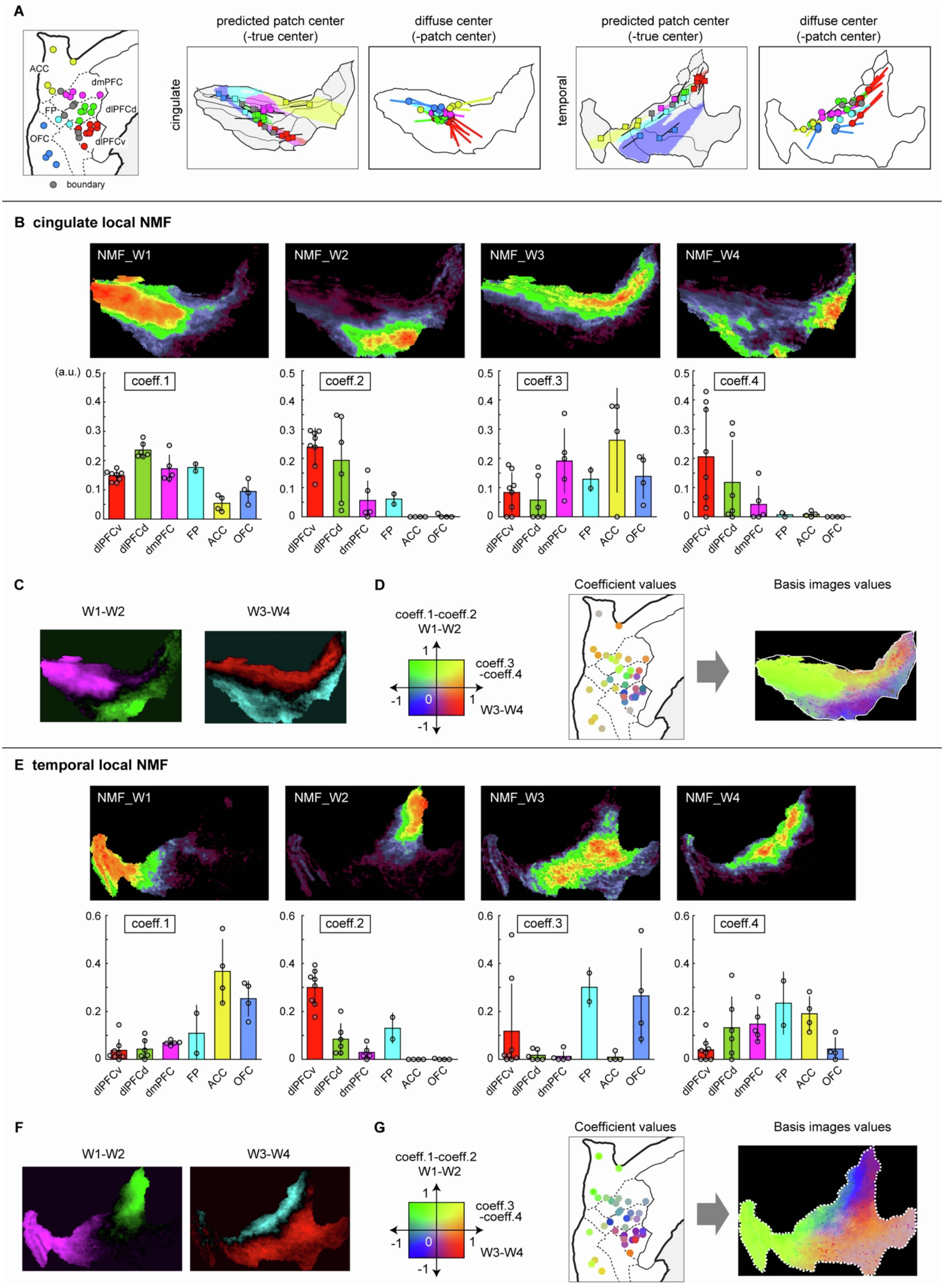
A
predicted patch center
(-true center)
diffuse center
(-patch center)
cingulate
temporal
ACC
dmPFC
FP
dlPFCd
OFC
dlPFCv
boundary
B cingulate local NMF
NMF_W1
NMF_W2
NMF_W3
NMF_W4
(a.u.)
coeff.1
coeff.2
coeff.3
coeff.4
dlPFCv
dlPFCd
dmPFC
FP
ACC
OFC
C
W1-W2
W3-W4
D
coeff.1-coeff.2
W1-W2
coeff.3
-coeff.4
W3-W4
Coefficient values
Basis images values
E temporal local NMF
F
G

Figure S9, Related to Figure 3: Local gradients revealed by NMF analysis of diffuse projections. (A) Comparison of the center of mass for the columnar patches and diffuse projections in the cingulate and temporal fields. The left-side panels are identical to those in Figure 2H and indicate the predicted positions of the patch centers for each injection together with deviation from the true center shown by bars. The right-side panels indicate the center of mass for the diffuse projections (log_{10} values) with deviation from the patch center shown by bars. Although less robust than the NMF analyses (see below), this analysis suggests the presence of a similar gradient to that of the columnar patches. (**B-D)** Local gradients within the cingulate field shown by the local NMF analysis. The basis images (W1-W4) and coefficients (coeff.1-4) are shown. **(C)** A combination of four basis images implicating local gradients. **(D)** Color indexing strategy to correlate injection coordinates and projection targets, as in Figure 3. (**E-G)** Local gradients within the temporal field shown by the local NMF analysis. The basis images (W1-W4) and coefficients (coeff.1-4) are shown. **(F)** A combination of four basis images implicating local gradients. **(G)** Color indexing strategy to correlate injection coordinates and projection targets, as in Figure 3.

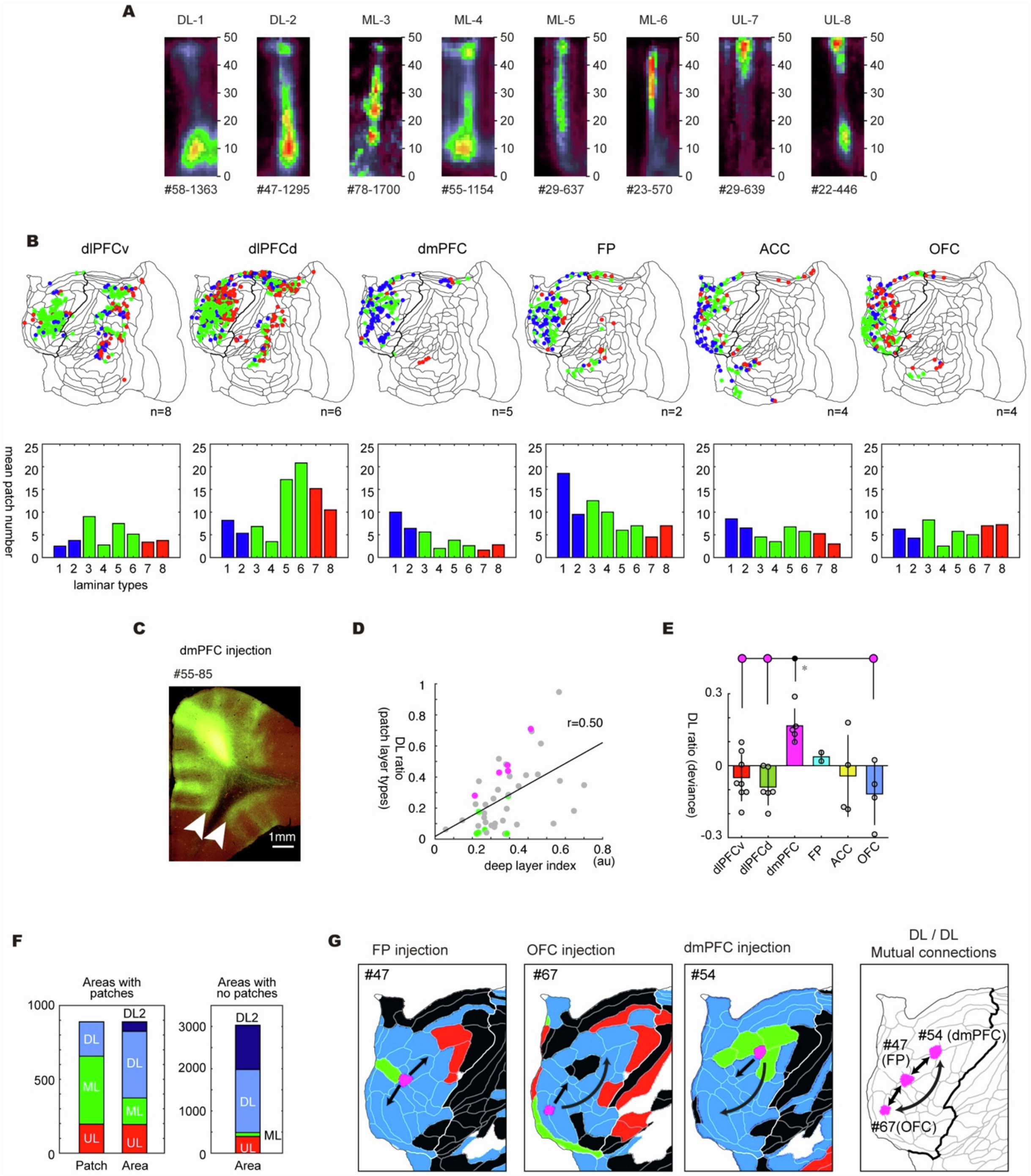

A
DL-1
DL-2
ML-3
ML-4
ML-5
ML-6
UL-7
UL-8
#58-1363
#47-1295
#78-1700
#55-1154
#29-637
#23-570
#29-639
#22-446
B
dlPFCv
dlPFCd
dmPFC
FP
ACC
OFC
n=8
n=6
n=5
n=2
n=4
n=4
mean patch number
laminar types
C
dmPFC injection
#55-85
1mm
D
DL ratio (patch layer types)
r=0.50
deep layer index
(au)
E
DL ratio (deviance)
dlPFCv
dlPFCd
dmPFC
FP
ACC
OFC
F
Areas with patches
Areas with no patches
DL2
DL
ML
UL
Patch
Area
G
FP injection
OFC injection
dmPFC injection
#47
#67
#54
DL / DL
Mutual connections
#47 (FP)
#54 (dmPFC)
#67(OFC)

Figure S10, Related to Figure 4: Laminar analyses of the columnar patches and the area-averaged diffuse projections. (A) Representative laminar patterns for the eight clusters shown in Figure 4A. **(B)** Areal distribution of three lamina types (DL: blue, ML: green, UL: red) for each PFC subregion and the bar graphs for eight clusters. The colors of the bar graphs indicate the DL, ML, and UL types. The areal distribution shows the overlay of all samples, and the bar graphs show the mean values per injection. **(C-E)** Evaluation of the influence of injection depth on the laminar patterns. **(C)** A coronal section view of a dmPFC sample showing widespread axonal projections in layer 6 (white arrowhead), which may contribute to the high DL ratio. **(D)** A scatter plot showing a weak correlation between the injection depth and patch lamina type. The DL ratios of the columnar patches were determined by numbers of DL-type patches among all the patches for each injection. The magenta and green dots correspond to injections into dmPFC and dlPFCd, respectively. **(E)** Subdivision differences of the layer type distribution. The influence of injection depth difference was adjusted based on correlation values shown in panel (D). Even after correction, the deviance in DL ratio showed a significant difference between dmPFC and dlPFCv, dlPFCd and OFC. **(F)** (Left panel) Comparison of dominant lamina types in the areas having patches with the area-averaged lamina types. (Right panel) Area-averaged lamina types in the areas with no columnar patches. **(G)** An example of area-wise reciprocal connections showing DL types in both directions. A part of area-averaged lamina maps was shown for each of the three injections into FP (#47), OFC (#67), and dmPFC (#54).

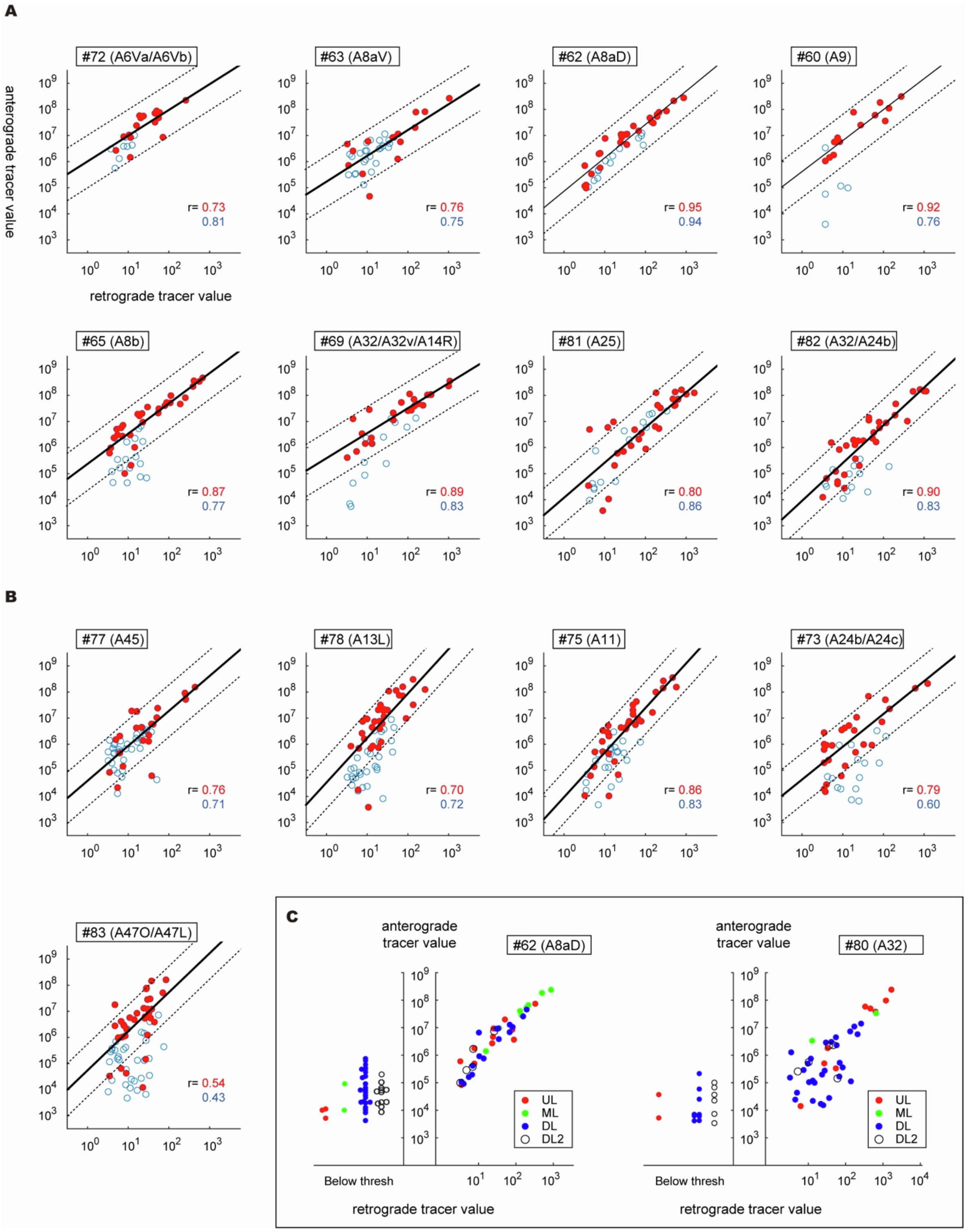

Figure S11, Related to Figure 5: Area-based comparison of anterograde and retrograde signals measured in log scale. (A) Samples with high-quality retrograde signals are shown (see method for the criteria). **(B)** Samples with noisy retrograde signals. These samples had scattered signals throughout the cortical areas that appeared likely to be false positives based on visual inspection of the morphology of the signals. We still observed moderate signal correlations, especially for the strong signals. The retrograde signals for #83 largely consisted of noise artifacts by visual inspection. **(C)** Two examples of anterograde-retrograde correlation in each area with laminar types assigned. Unlike in panels (A) and (B) and Figure 5G, areas with below-threshold retrograde signals are indicated on the left side of the panel. As these examples show, DL (blue dot) and DL2 (open circle) types tended to be associated with low anterograde and retrograde signals.

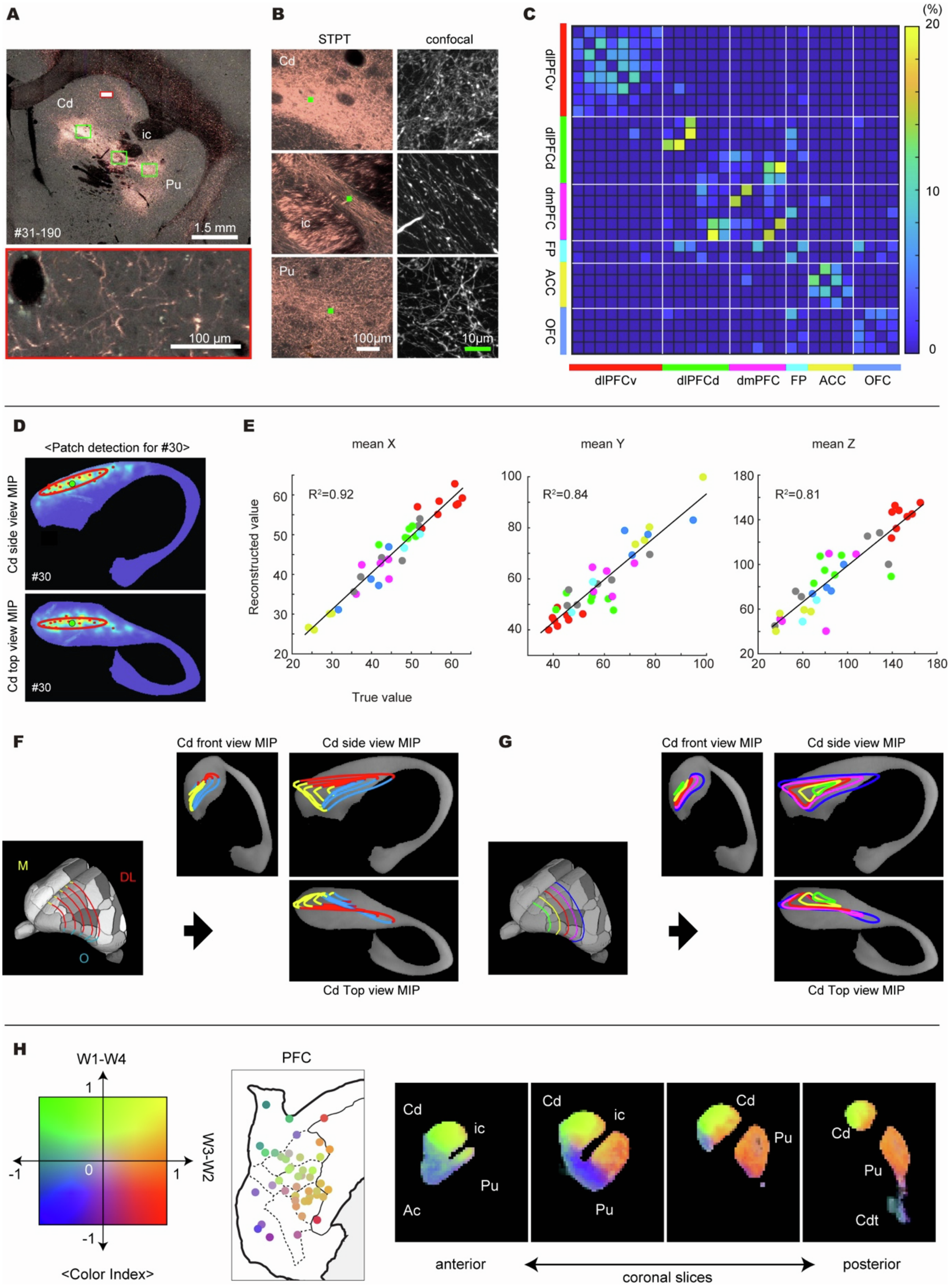

A
Cd
ic
Pu
#31-190
1.5 mm
100 μm
B
STPT
confocal
Cd
ic
Pu
100μm
10μm
C
(%)
20
10
0
dlPFCv
dlPFCd
dmPFC
FP
ACC
OFC
D
<Patch detection for #30>
Cd side view MIP
Cd top view MIP
#30
E
mean X
mean Y
mean Z
Reconstructed value
True value
R²=0.92
R²=0.84
R²=0.81
F
M
DL
O
Cd front view MIP
Cd side view MIP
Cd Top view MIP
G
H
W1-W4
W3-W2
<Color Index>
PFC
Cd
ic
Pu
Ac
Cdt
anterior
coronal slices
posterior

Figure S12, Related to Figure 6: Patchy (focal) and diffuse corticostriatal projections. (A) An example of the original section image of the corticostriatal projections. Here, the fluorescence of the tracer signals is colored pink. The white rectangle with red contours is magnified below as an example of a sparsely innervated region. The three green rectangles are magnified on panel (B). **(B)** A clump of dense tracer signals in Cd, a bridge region in the internal capsule, and a dense region in Pu are shown magnified. The green-filled rectangles in the STPT images indicate the approximate positions for confocal microscopy on the right panels. **(C)** The colocalization of binarized tracer signals between two samples within and across six PFC subregions. See Figure 6C for example images, and Figure 6E for the averaged values. The average rate of colocalization was generally higher within the same PFC subregion than between different subregions (Figure 6E), except that some dlPFCd samples showed similar distributions to dmPFC samples. Overall, however, the colocalization rate was low even in the same subregions. **(D)** Detection of local maxima of tracer convergence patch (red dots); the centroid of these patches is indicated by a green dot. The distribution of these patches could generally be well approximated by an elongated ellipsoid (red ellipses). The MIP side view and top view of the left caudate nucleus are shown. **(E)** Scatter plots comparing the true and reconstructed values from polynomial regression models based on injection coordinates. Six core PFC subregions and border injections are used for regression fitting. **(F)** Topographic projections from the frontal areas to the caudate nucleus predicted by the polynomial regression model. Five contours covering the dorsolateral (DL; red), orbital (O; blue), and medial (M; yellow) sides of the frontal cortex are projected onto the caudate nucleus. **(G)** The five contours colored green, yellow, orange, red, and blue from the rostral to the caudal end are projected onto the caudate nucleus. **(H)** NMF components and coefficients of the diffuse projections were color-coded in a similar manner to that used for Figure 3H. The coronal sections roughly correspond to AP+12.5, AP+11.5, AP+9.0, and AP+7.0 in the Paxinos atlas [S9]. (https://www.marmosetbrain.org/reference). Ac, nucleus accumbens; Cd, caudate nucleus; Cdt, tail of caudate nucleus; Pu, putamen; ic, internal capsule.

Data ID	Brain/MINDS ID	age (y)	sex	subregion	area	Core 6 PFC	Injection Volume(mm^3)	Deep Layer index
#40	R01_0061	8.5	M	PM/dmPFC	A8b/A6DM		3.37	0.67
#56	R01_0083	6	F	PM	A6DR		2.51	0.24
#57	R04_0079	4.3	F	PM	A6DM		3.92	0.34
#72	R01_0104	5.3	M	PM	A6Va/A6Vb		1.47	0.31
#44	R01_0069	6.6	F	PM	A6DR		2.48	0.38
#45	R01_0070	6.6	F	PM/dlPFCv	A8aV/A6Va		3.11	0.06
#49	R01_0076	8.9	M	dlPFCv	A8aV	o	1.85	0.32
#63	R01_0091	2.4	M	dlPFCv	A8aV	o	1.77	0.34
#36	R01_0057	4.1	F	dlPFCv	A8aV	o	2.35	0.23
#42	R01_0063	8.3	M	dlPFCv	A8aV	o	3.22	0.4
#46	R01_0075	5.6	F	dlPFCv	A8aV	o	4.49	0.3
#22	R01_0029	5	F	dlPFCv	A8aV	o	1.34	0.27
#28	R01_0040	5.6	M	dlPFCv	A8aV	o	0.31	0.38
#43	R01_0064	8.3	M	dlPFCv/vlPFC	A8aV/A47L		3.22	0.75
#77	R01_0110	6.7	F	dlPFCv	A45	o	1.71	0.15
#23	R01_0030	5.3	F	vlPFC	A47L/A47M		1.21	0.39
#29	R01_0043	3.3	F	dlPFCd	A8aD	o	1.39	0.27
#31	R01_0048	2.4	F	dlPFCd	A8aD	o	3.34	0.39
#62	R01_0088	3.7	F	dlPFCd	A8aD	o	3.16	0.23
#19	R01_0026	5	F	dlPFCd	A8aD	o	1.28	0.39
#21	R01_0028	5.1	M	dlPFCd/FP	A10/A46D		1.29	0.36
#30	R01_0046	3.4	M	dlPFCd	A8aD	o	2.52	0.24
#33	R01_0054	8.1	F	dlPFCd/dmPFC	A8aD/A9/A8b		3.14	0.52
#38	R01_0059	8.1	M	dlPFCd	A8aD	o	3.73	0.22
#41	R01_0062	8.5	M	dlPFCd/dmPFC	A8aD/A9		3	0.26
#48	R01_0071	7.8	M	dmPFC	A9	o	2.16	0.39
#60	R01_0094	10.8	M	dmPFC	A9	o	1.37	0.21
#55	R01_0080	7.4	F	dmPFC	A9/A8b	o	3.97	0.39
#65	R04_0095	2.6	F	dmPFC	A8b	o	2.8	0.51
#54	R01_0081	7.6	F	dmPFC	A8b	o	2.34	0.34
#47	R01_0052	6.2	F	FP	A46V/A10/A47L	o	2.66	0.49
#58	R04_0080	3.2	F	FP	A10	o	4.22	0.45
#50	R01_0072	10.4	F	ACC	A32	o	2.51	0.44
#80	R01_0112	6	F	ACC	A32/A9		3.16	0.22
#64	R01_0092	2.4	M	ACC	A24a	o	1.07	0.8
#69	R01_0095	2.3	M	ACC	A32/A32v/A14R	o	5	0.57
#81	R01_0114	9.8	F	ACC	A25	o	6.97	0.39
#52	R01_0078	8.9	F	OFC	A13L/A13M	o	1.64	0.55
#67	R01_0090	2.9	F	OFC	A13M/A13L	o	0.66	0.67
#78	R01_0107	2.5	M	OFC	A13L	o	2.92	0.28
#75	R04_0058	5	F	OFC	A11	o	0.51	0.64
#73	R01_0098	3.3	M	dACC	A24b/A24c		3.08	0.26
#82	R01_0115	8.4	F	dACC	A32/A24b		3.07	0.27
#83	R04_0023	5.2	M	vlPFC	A47O/A47L		0.59	0.32

Supplementary Table1, Related to STAR Methods.

Marmoset injections analyzed in the present study.

Supplementary References

S1. Eldred, G.E., Miller, G.V., Stark, W.S., and Feeney-Burns, L. (1982). Lipofuscin: resolution of discrepant fluorescence data. Science *216*, 757–759. 10.1126/science.7079738.

S2. Theodoni, P., Majka, P., Reser, D.H., Wójcik, D.K., Rosa, M.G.P., and Wang, X.-J. (2021). Structural Attributes and Principles of the Neocortical Connectome in the Marmoset Monkey. Cereb Cortex *32*, 15–28. 10.1093/cercor/bhab191.

S3. Markov, N.T., Misery, P., Falchier, A., Lamy, C., Vezoli, J., Quilodran, R., Gariel, M.A., Giroud, P., Ercsey-Ravasz, M., Pilaz, L.J., et al. (2011). Weight consistency specifies regularities of macaque cortical networks. Cereb Cortex *21*, 1254–1272. 10.1093/cercor/bhq201.

S4. Ercsey-Ravasz, M., Markov, N.T., Lamy, C., Van Essen, D.C., Knoblauch, K., Toroczkai, Z., and Kennedy, H. (2013). A predictive network model of cerebral cortical connectivity based on a distance rule. Neuron *80*, 184–197. 10.1016/j.neuron.2013.07.036.

S5. Iriki, A., Okano,J., H., Sasaki, E., and Okano, H. (2018). The 3-dimensional atlas of the marmoset brain (Springer Berlin Heidelberg).

S6. Woodward, A., Hashikawa, T., Maeda, M., Kaneko, T., Hikishima, K., Iriki, A., Okano, H., and Yamaguchi, Y. (2018). The Brain/MINDS 3D digital marmoset brain atlas. Sci Data *5*, 180009. 10.1038/sdata.2018.9.

S7. Xu, R., Bichot, N.P., Takahashi, A., and Desimone, R. (2022). The cortical connectome of primate lateral prefrontal cortex. Neuron *110*, 312-327.e7. 10.1016/j.neuron.2021.10.018.

S8. Levitt, J.B., Lewis, D.A., Yoshioka, T., and Lund, J.S. (1993). Topography of pyramidal neuron intrinsic connections in macaque monkey prefrontal cortex (areas 9 and 46). J Comp Neurol *338*, 360–376. 10.1002/cne.903380304.

S9. Paxinos, G., Watson, C., Petrides, M., Rosa, M., and Tokuno, H. (2012). The marmoset brain in stereotaxic coordinates (Academic Press).
